# BOLD Monitoring in the Neural Simulator ANNarchy

**DOI:** 10.3389/fninf.2022.790966

**Published:** 2022-03-22

**Authors:** Oliver Maith, Helge Ülo Dinkelbach, Javier Baladron, Julien Vitay, Fred H. Hamker

**Affiliations:** Department of Computer Science, Chemnitz University of Technology, Chemnitz, Germany

**Keywords:** blood-oxygen-level-dependent signal, neural simulator, spiking networks, rate-coded networks, Balloon model, neurovascular coupling, cerebral blood flow, cerebral metabolic rate of oxygen

## Abstract

Multi-scale network models that simultaneously simulate different measurable signals at different spatial and temporal scales, such as membrane potentials of single neurons, population firing rates, local field potentials, and blood-oxygen-level-dependent (BOLD) signals, are becoming increasingly popular in computational neuroscience. The transformation of the underlying simulated neuronal activity of these models to simulated non-invasive measurements, such as BOLD signals, is particularly relevant. The present work describes the implementation of a BOLD monitor within the neural simulator ANNarchy to allow an on-line computation of simulated BOLD signals from neural network models. An active research topic regarding the simulation of BOLD signals is the coupling of neural processes to cerebral blood flow (CBF) and cerebral metabolic rate of oxygen (CMRO2). The flexibility of ANNarchy allows users to define this coupling with a high degree of freedom and thus, not only allows to relate mesoscopic network models of populations of spiking neurons to experimental BOLD data, but also to investigate different hypotheses regarding the coupling between neural processes, CBF and CMRO2 with these models. In this study, we demonstrate how simulated BOLD signals can be obtained from a network model consisting of multiple spiking neuron populations. We first demonstrate the use of the Balloon model, the predominant model for simulating BOLD signals, as well as the possibility of using novel user-defined models, such as a variant of the Balloon model with separately driven CBF and CMRO2 signals. We emphasize how different hypotheses about the coupling between neural processes, CBF and CMRO2 can be implemented and how these different couplings affect the simulated BOLD signals. With the BOLD monitor presented here, ANNarchy provides a tool for modelers who want to relate their network models to experimental MRI data and for scientists who want to extend their studies of the coupling between neural processes and the BOLD signal by using modeling approaches. This facilitates the investigation and model-based analysis of experimental BOLD data and thus improves multi-scale understanding of neural processes in humans.

## 1. Introduction

Network models are simulated neural networks composed of multiple computational units that model the dynamics of biological neurons at various levels of complexity: macroscopic mean-field or neural mass models simulate the average dynamics of large groups of neurons, rate-coded point neuron models simulate the instantaneous mean firing rate of individual neurons, spiking point neuron models simulate precise spike timings, while multi-compartmental neuron models also consider the 3D structure of the neurons. Such network models can exhibit complex dynamics due to the recurrent connectivity between the simulated neurons and can be validated against a large amount of experimental data and make extensive predictions at different scales, such as patterns in spike timing, local field potentials or electroencephalography, and blood-oxygen-level-dependent (BOLD) signals from magnetic resonance imaging (MRI). Large-scale network models are becoming increasingly common in computational neuroscience (see Einevoll et al., 2019 for a review about brain simulations with network models). Concerning MRI data, network models can be used primarily to examine the underlying neural mechanisms of the experimental non-invasive data or, for example, to better understand the relationship between the structural connectivity and the functional dynamics of neural circuits (Popovych et al., 2019).

The ANNarchy neural simulator (Vitay et al., 2015) provides a user-friendly equation-based interface which can be used to create large-scale rate-coded and spiking network models at different levels of biological realism. Recently, the ANNarchy neural simulator has been combined with the whole-brain neural simulator The Virtual Brain (TVB) (Ritter et al., 2013; Sanz Leon et al., 2013; Meier et al., 2021) to allow the creation of multi-scale network models. This allows to study how processes in detailed spiking network models of specific brain regions such as the basal ganglia created in ANNarchy affect the dynamics of the whole cortex simulated in TVB (Meier et al., 2021). To further improve the usability of ANNarchy, we introduce a BOLD signal monitoring module (called BOLD monitor in ANNarchy) that allows obtaining simulated BOLD signals from spiking and rate-coded network models in an on-line manner.

Several modeling tools already provide utilities to obtain simulated BOLD signals from network models TVB, Dynamic Causal Modeling (Friston et al., 2003) in SPM (Penny et al., 2011), neuRosim (Welvaert et al., 2011), which so far have been applied mainly to network models at the macroscopic level of detail (Vanni et al., 2015). These methods mainly use variants of the Balloon model to compute simulated BOLD signals (Buxton et al., 1998, 2004; Stephan et al., 2007). Hereafter, we will refer to the Balloon model and other such models that convert an input time signal into a simulated BOLD signal, generally as BOLD models. A critical open issue when simulating BOLD signals from network models is the neurovascular coupling, i.e., which neural mechanisms are associated with the metabolism and dynamics of the blood vessels that ultimately cause the BOLD signal. This is essential information needed to meaningfully couple a network model with a BOLD model. The issue of the neurovascular coupling remains unsolved and is an active area of research (Vanni et al., 2015; Buxton, 2021; Howarth et al., 2021). Recently, it has been proposed that cerebral blood flow (CBF) and cerebral metabolic rate of oxygen (CMRO2) may be driven separately by distinct neural processes (Buxton, 2012, 2021). As these variations are not captured by the classic Balloon model implementations in current tools, researchers need more flexible tools that allow them to define their own BOLD models.

The neural simulator ANNarchy is primarily concerned with models ranging from the mesoscopic to the microscopic level that simulate biological neurons as single units and can thus account for more detailed processes, which can include different ionic membrane currents and account for the dynamics of specific classes of real neurons (Humphries et al., 2009; Corbit et al., 2016; Goenner et al., 2021). Thus, ANNarchy allows to consider various neural processes for the implementation and investigation of neurovascular coupling. The BOLD monitor not only allows linking predefined BOLD models (e.g., the Balloon model variants, Stephan et al., 2007) to a rate-coded or spiking network model but also gives the user freedom in defining the neurovascular coupling and the BOLD model itself, allowing to investigate different hypotheses regarding the link between neural processes and BOLD signals.

In this article, we present the rationale, implementation and use of the BOLD monitor in ANNarchy. We first demonstrate the use of the classic Balloon model as a BOLD model for the BOLD monitor. We then demonstrate how to create a user-defined BOLD model. Finally, using a simple network model as an example, we demonstrate how the BOLD monitor can be used to compare various hypotheses about neurovascular coupling in simulation.

## 2. The Balloon Model

### 2.1. The Classic Balloon Model

The Balloon model was originally designed by Buxton et al. (1998). It describes the changes in the BOLD signal of a tissue region, often called region of interest (ROI), as a function of normalized CBF (*f*_*in*_). According to this model, the BOLD signal corresponds to the sum of the extravascular and intravascular signal resulting from the normalized total deoxyhemoglobin content (*q*) and the normalized venous volume fraction (*v*). The normalized venous volume fraction is described as a balloon that expands with increasing inflow and slowly recovers after a stimulus. The normalized deoxyhemoglobin content is determined by the dynamics of the volume fraction and the blood oxygen extraction fraction (*E*), whose behavior is based on the oxygen limitation model (Buxton and Frank, 1997).

**Figure 1:**
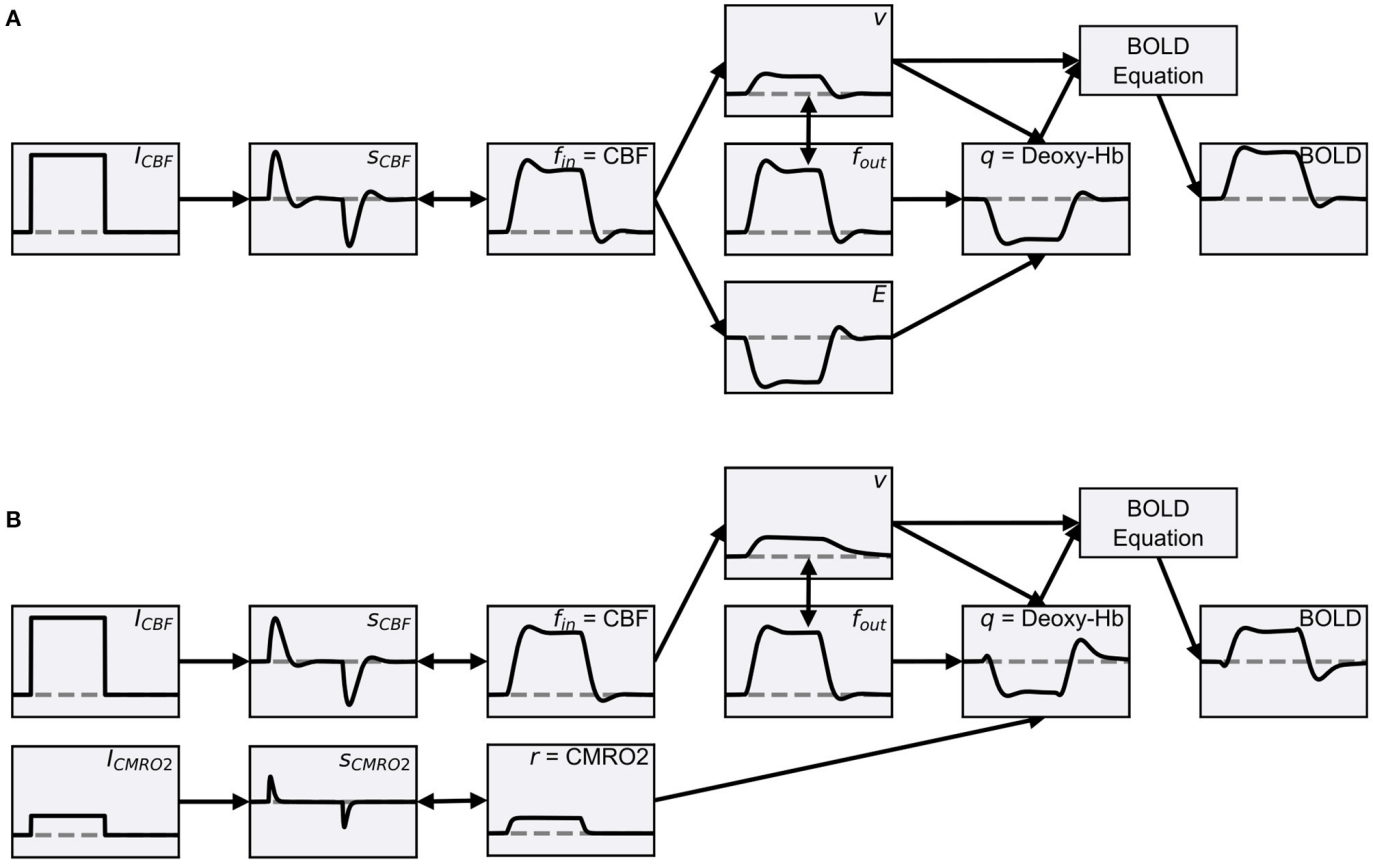
**(A)** Schematic overview of the classic Balloon model (Buxton et al., 1998) with the neurovascular coupling extension of Friston et al. (2000). The data was simulated using ANNarchy's default BOLD monitor which recorded a single artificial neuron, whose activity (the source variable for the BOLD monitor) was manually set. The activity of the neuron and thus the input signal (*I*_*CBF*_) were manually increased from zero to 0.2 for 20 s. The normalized CBF (*f*_*in*_) changes as a function of the CBF-driving signal (*s*_*CBF*_), which is subject to negative feedback from itself and *f*_*in*_. *f*_*in*_ is coupled to the blood oxygen extraction fraction (*E*) and increases the normalized volume fraction of the venous compartment (*v*), which behaves like a balloon and decreases with outflowing blood (*f*_*out*_). The normalized total deoxyhemoglobin content (*q*) increases by oxygen extraction of inflowing blood and decreases with outflowing deoxyhemoglobin-containing blood. Finally, relative changes of the BOLD signal are calculated. The gray horizontal lines correspond to one for the quantities normalized to their baseline in the Balloon model (*f*_*in*_, *f*_*out*_, *v*, *q*). For *I*_*CBF*_, *s*_*CBF*_ and *BOLD* the gray horizontal lines correspond to zero and for *E* to *E*_0_. **(B)** Balloon model with parallel driven CBF and CMRO2. Instead of coupling the increase in *q* with *f*_*in*_ *via* *E*, the normalized CMRO2 (*r*) is used directly (see Buxton et al., 2004), which is driven by a second input signal (*I*_*CMRO*2_) like the normalized CBF (increased to 0.05 for 20 s). Further, *f*_*out*_ is described by the equations of Buxton et al. (2004) which causes *v* to decrease slower. Besides these changes, the processes are the same as in **(A)** and the plots of the same quantities have the same limits in **(A,B)**. The equations of both models can be found in Supplementary Sections 2, 4.2.

Friston et al. (2000) extended the Balloon model so that it can be used to simulate BOLD signals using network models. The extension included a neurovascular coupling component that links the normalized CBF of the Balloon model to simulated neuronal activity. Based on this extension, the normalized CBF is modeled as a damped oscillator that is stimulated by neuronal activity. This extension allows the Balloon model to be used to simulate a change in the BOLD signal due to a change in some type of simulated neuronal activity (hereafter, more generally referred to as input signal). In this form, the Balloon model has been used in several studies to compute simulated BOLD signals from network models (Friston et al., 2003; Smith et al., 2011; Deco and Jirsa, 2012; Van Hartevelt et al., 2014; Bennett et al., 2015; Maith et al., 2021). The individual components of the extended Balloon model and their dynamics following a rectangular input signal change are shown in Figure 1A.

Different values for the parameters and even variations of some equations of the model can be found in the literature. We use a version from Stephan et al. (2007) with a non-linear BOLD equation with revised coefficients for our default BOLD monitor. The other versions of Stephan et al. (2007) are also implemented in ANNarchy and available as alternatives. All equations are summarized in Supplementary Section 2. The implementation of the default model in ANNarchy is described in Section 3.4.

### 2.2. The Two-Input Balloon Model

In the classic Balloon model, CBF and CMRO2 are tightly coupled. The greater increase in CBF compared to CMRO2 in response to a stimulus is explained by the oxygen limitation model (Buxton and Frank, 1997). This model is based on the assumptions that oxygen coming from the capillaries is completely metabolized in the tissue and that all brain capillaries are perfused at rest. As a consequence, an increase in CMRO2 would only be possible by increasing the transport of oxygen from the capillaries to the tissue, and an increase in CBF would be accompanied by an increase in capillary blood velocity. Because an increase in CBF increases the available oxygen in the capillaries, but also decreases the fraction of oxygen extracted from the capillaries, an increase in CMRO2 (i.e., oxygen transport from the capillaries to the tissue) requires a disproportionate increase in CBF (for further details, see Buxton and Frank, 1997).

However, in recent years, it has been proposed that CBF and CMRO2 are driven in parallel by different sources rather than being tightly coupled (Buxton, 2012, 2021; Buxton et al., 2014). Recently, Buxton (2021) has put forward a new theory, based on the thermodynamics of metabolism, that could explain why CBF needs to increase more than CMRO2 in response to a stimulus and has proposed that CBF and CMRO2 are both driven in parallel in a feed-forward manner. The open question here is by which neural signals CBF and CMRO2 are driven. One suggestion is that CMRO2 is tightly coupled to the energy consumption of neurons, whereas CBF is controlled by vasodilatory signals. These vasodilatory signals are not necessarily coupled to energy consumption and are caused, for example, by activated astrocytes (Buxton, 2012; Howarth et al., 2021). Network models, in which a wide variety of populations can be simulated and manipulated in a controlled manner, may be useful in investigating this question. Therefore, not only the classic Balloon model with tightly coupled CBF and CMRO2 can be used in our BOLD monitor, but also user-defined BOLD models, potentially using more than one input signal from the network model.

We demonstrate how to define BOLD models with multiple input signals for the BOLD monitor in ANNarchy by implementing a modified version of the Balloon model where CBF and CMRO2 are driven in parallel by separate input signals (hereafter referred to as two-input Balloon model). For simplicity, in the two-input Balloon model, we describe both, the normalized CBF and CMRO2, as damped oscillators similar to the normalized CBF in the classic Balloon model version of Friston et al. (2000). Equal input signals elicit responses with equal amplitudes for the normalized CBF and CMRO2. Thus, the coupling between CBF and CMRO2 is determined by the coupling of the two input signals. Figure 1B demonstrates how the individual components of the two-input Balloon model change during stimulation. Compared to the normalized CBF, the normalized CMRO2 responds faster to a changing input signal and without an overshoot or undershoot. The faster response allows for an initial dip in the BOLD signal. For the transformation from normalized CBF and CMRO2 to *q* and *v*, we use the Balloon model equations from Buxton et al. (2004). This is a slightly modified version of the classic Balloon model, which additionally considers viscoelastic effects causing the venous volume fraction to lag behind its steady-state relation with the outflow during transient changes. Thus, a post-stimulus undershoot in the BOLD signal is caused by the undershoot of the CBF (based on the damped oscillator modeling approach) as well as by the slow recovery of the venous volume fraction (based on the viscoelastic effects). Finally, the change in the BOLD signal is computed by the non-linear BOLD equation with revised coefficients from Stephan et al. (2007). A more detailed description including the equations of the two-input Balloon model summarized here can be found in Supplementary Section 4.2.

## 3. BOLD Monitor

### 3.1. ANNarchy Neural Simulator

The ANNarchy neural simulator is intended for the simulation of network models at the single-unit level using rate-coded and spiking neuron models. The equation-based interface of ANNarchy allows a flexible and easy implementation of network models by defining equations describing the dynamics of specific neuron types in so called neuron models and equations defining synaptic transmission dynamics (e.g., plasticity) in so called synapse models (Vitay et al., 2015). For efficiency, the model description is transformed into optimized C++ code, optionally using parallel programming frameworks such as openMP for multi-core CPUs or CUDA for GPUs (Dinkelbach et al., 2019). An earlier version of the BOLD monitor in ANNarchy relied on the normalization of pre-synaptic activity and was used in Maith et al. (2021). This implementation was limited to one specific BOLD model and allowed only a few parameter variations, unlike the version presented here. All the simulations in this work use the version 4.7.0.1 of the neural simulator ANNarchy. All references to neurons, populations, synapses, BOLD signals and other neural quantities and data in the following sections refer to simulated values from a network model.

### 3.2. General Concept

**Figure 2:**
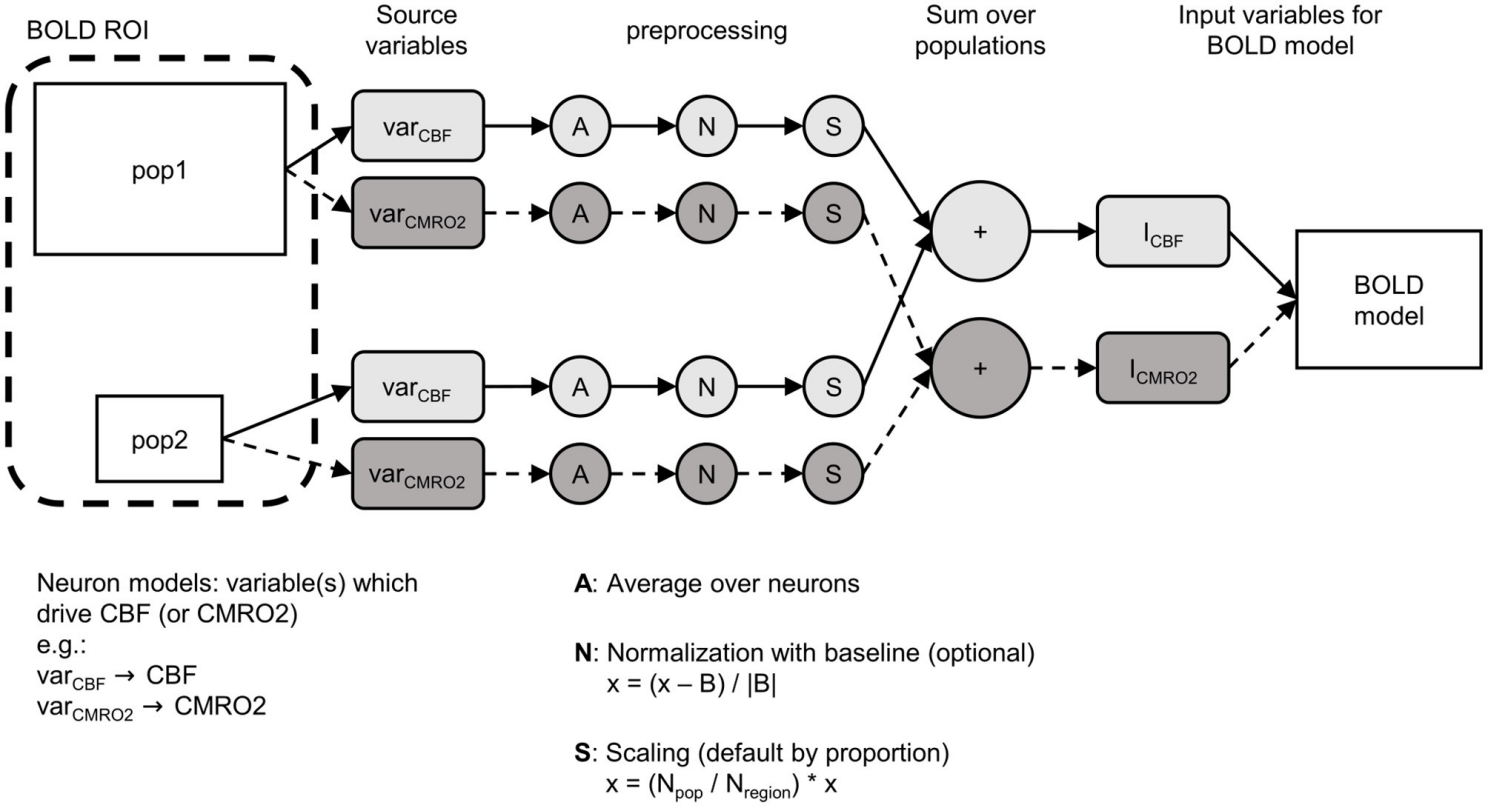
Schematic overview of how the BOLD monitor calculates the input signals (here I_CBF_, I_CMRO2_) for a BOLD model (e.g., Balloon model). The ROI from which the BOLD signal should be calculated can consist of multiple populations (here pop1, pop2). In the neuron models of each population, the variables (var_CBF_, var_CMRO2_) have to be defined, which are used to calculate the input signals (referred to as source variables). Three preprocessing steps are applied to the source variables per population: (1) averaging over all neurons of the population, (2) optional normalization, (3) scaling by proportion of the population in the ROI. Finally, the signals resulting from the preprocessed source variables are summed across all populations and fed into the BOLD model as input signals. x, the averaged signal; B, baseline; N_pop_, size of the population; N_region_, total number of neurons in the ROI.

BOLD models, for example the Balloon model (Buxton et al., 1998) or the Davis model (Davis et al., 1998), are based on signals that characterize the dynamics of an entire ROI, such as the change in the normalized CBF or CMRO2 (Figure 2, *f*_*in*_, *r*). To combine such a BOLD model with a network model, it is necessary to bridge the gap between these ROI-wide signals and the individual components of the ROI in the network model (e.g., multiple populations, individual neurons). In this section, we will focus on the processes necessary to obtain the input signals for a BOLD model from a ROI that represents part of a network model consisting of multiple populations. Figure 2 shows the general functionality of the BOLD monitor in ANNarchy.

First, the populations of the network model that are part of the ROI for the BOLD computation have to be specified and instantiated. In the example shown in Figure 2, the ROI consists of two populations labeled pop1 and pop2. From the definition of their neuron models, variables must be selected or defined (hereafter referred to as source variables) which will be used to derive the input signals of the BOLD model. In Figure 2, two different source variables are defined: one variable *var*_*CBF*_ that causes the input signal of the CBF (*I*_*CBF*_) and one variable *var*_*CMRO2*_ that causes the input signal of the CMRO2 (*I*_*CMRO2*_). These source variables can correspond to any variables or combinations of variables present in the neuron models (membrane potential, firing rate, etc.).

After defining the ROI and the mapping between source variables in the neuron models and the input variables of the BOLD model, the BOLD monitor implements four preprocessing steps. First, the source variables are averaged over all the neurons for each population of the ROI, resulting in only one signal per population and source variable. This averaging is followed by an optional population-wide normalization that computes the relative deviation of the signal from a baseline value. The baseline corresponds to the mean of the raw averaged source variable signal calculated over a specified initial period. This normalization is useful when deviations from the resting-state are required as input signal in the BOLD model. After the optional normalization, the signals are scaled per population. By default, the signals of each population are scaled based on the ratio between the size of the population and the total number of neurons in the ROI. Thus, the larger a population, the greater its influence on the input variables of the BOLD model. Finally, the population signals are summed across all populations of the ROI, resulting in one input signal for each input variable of the BOLD model.

### 3.3. A Simple Example

This section describes a minimal example demonstrating the use of the BOLD monitor in the ANNarchy framework. ANNarchy modules and the BOLD extension must first be imported:


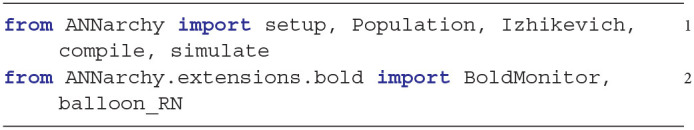


The evaluation of equations is performed with the forward Euler numerical method using a fixed time grid of step *dt* (in ms):


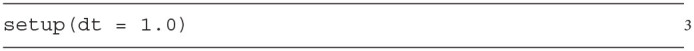


Two populations, both composed of 100 Izhikevich spiking neurons are then created (line 4, 5). The Izhikevich neuron model is part of the standard models pre-implemented in ANNarchy, with equations and parameters derived from Izhikevich (2003). Initially, the baseline activity in both populations is defined by setting their *noise* variables to 5.0 (line 7). The term *noise* refers to an internal variable of the pre-implemented Izhikevich neuron model in ANNarchy which simply determines a baseline current in the membrane potential equation.


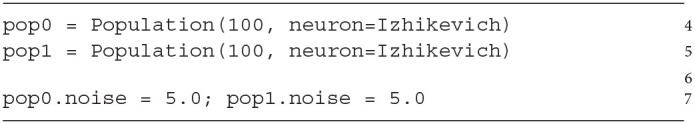


To keep the example simple and still have a modulation in the source variable of the BOLD monitor, the baseline activity (the *noise* variable) is varied during the simulation to mimic the effect of external inputs. The mean-firing rate *r* of the individual neurons is used as the source variable for the computation of the BOLD signal. As the computation of this value requires an additional overhead, it must be enabled explicitly. The time window for the averaged activity is set to 100 ms:


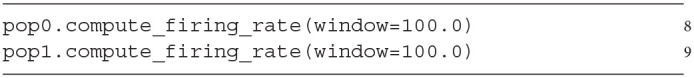


The BOLD monitor is then created and initialized (line 10–16). The populations in the ROI have to be assigned in the *populations* argument in form of a list of or a single population (line 11). The desired BOLD model can be optionally defined in the argument *bold_model* by assigning the corresponding BOLD model object (line 12). The BOLD model can be either one of the built-in BOLD models provided by the module or user-defined as we will demonstrate in Section 3.4. The default BOLD model is the built-in implementation *balloon_RN* containing the Balloon model with revised coefficients and a non-linear BOLD equation (described in Section 2, implementation shown in Section 3.4).

The mapping between the source variables of the populations (here mean-firing rate *r*) and the input signals of the BOLD model (referred to as input variables, here *I_CBF*) has to be defined in the *mapping* argument by providing a dictionary for each input variable-source variable pair (line 13).

A time window relevant to the normalization of the source variables can be optionally defined (in ms, line 14), whose purpose we explain in Section 4.2. By default, no normalization is performed.

Finally, the variables of the BOLD model which should be recorded during the simulation can be optionally assigned in the *recorded_variables* argument (line 15) as a string or list of strings. All variables of the BOLD model can be recorded. By default, the output variable of the BOLD model (here the variable *BOLD*) defined in the BOLD model implementation (see Section 3.4) is recorded.


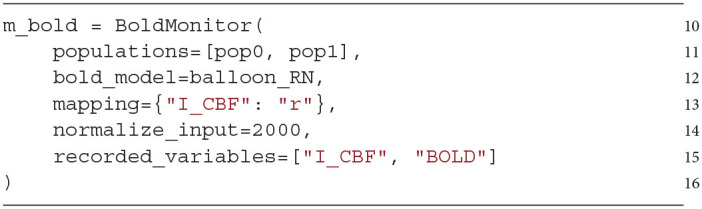


The C++ code representing the model (network model and BOLD monitor) can now be generated and compiled:


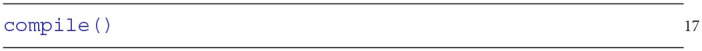


The last part of this section describes a sample simulation to demonstrate the BOLD recording on our simple example. A short simulation period (1,000 ms, line 19) ensures that the network reaches a stable state, which is necessary for a meaningful baseline calculation (required for the normalization outlined in Section 4.2). The recording of BOLD signals is started (line 22) and the simulation is run for 5 s (line 25). After this, the baseline activity (*noise* variable) of half of the recorded neurons (one population, pop0) is increased for 5 s (lines 26, 27) and afterwards set back to the previous value (line 28, 29).


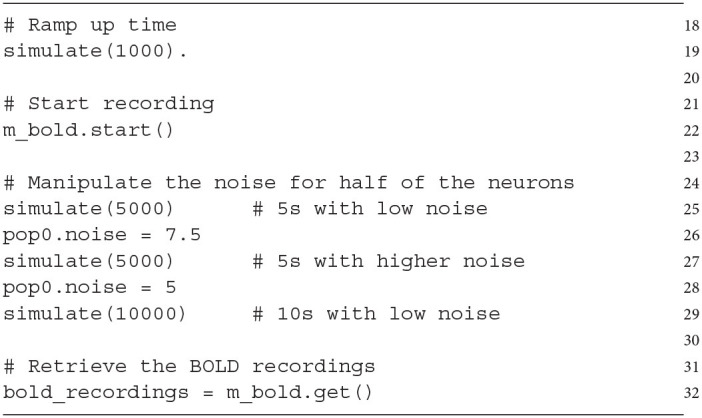


**Figure 3:**
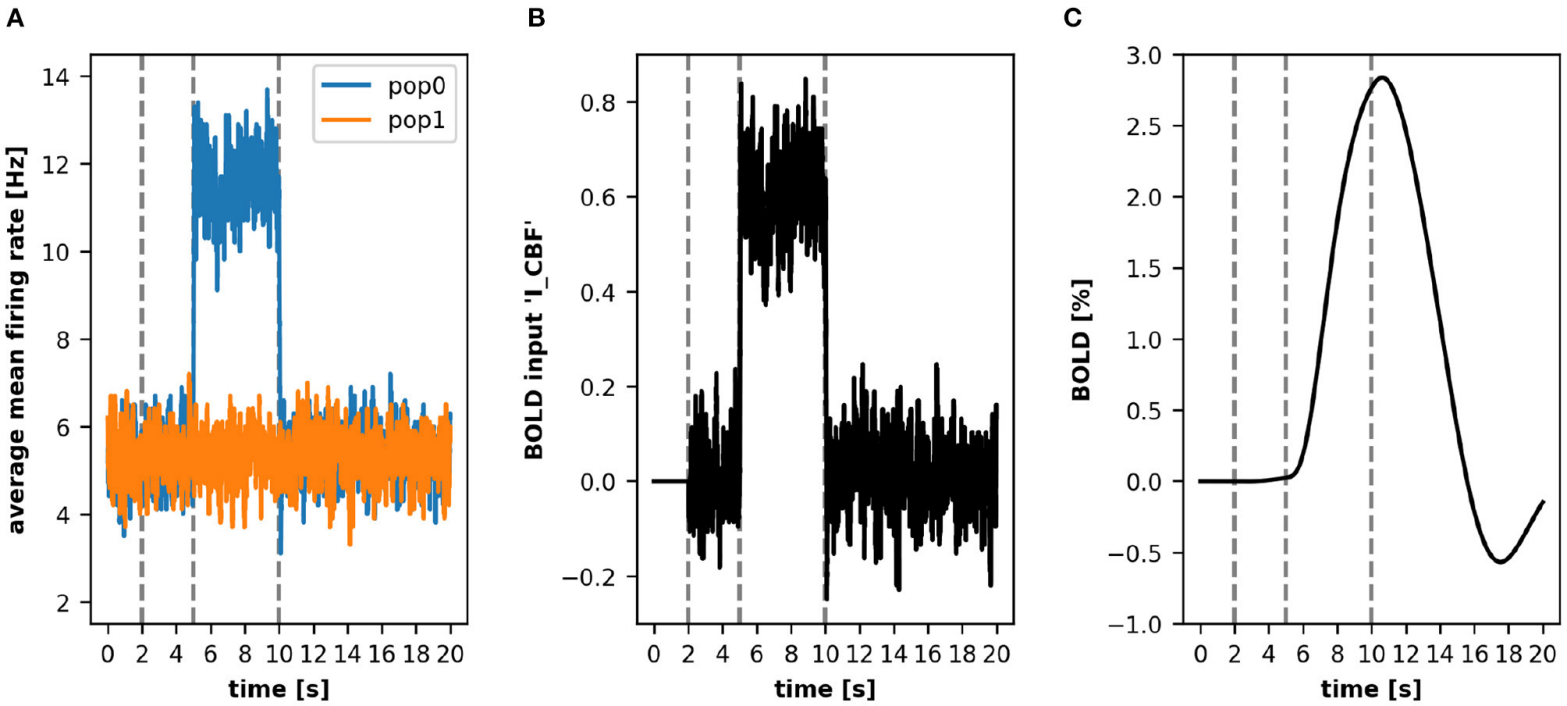
A simple simulation using the ANNarchy BOLD monitor. In this example, we obtain a BOLD signal from two populations *pop0* and *pop1*. Both populations contribute their mean-firing rate *r* **(A)** to the BOLD model which we defined in Section 3.4. After 5 s of simulation, the *noise* variable in *pop0* is increased which leads to a higher mean-firing rate. This increases the input signal *I_CBF* **(B)** and consequently the computed BOLD signal **(C)**. For clarity, the vertical lines depict three relevant time points (left to right): end of baseline period, time point of increased *noise* variable, time point of reset *noise* variable.

This leads to an increased mean firing rate in the recorded area and consequently to a BOLD signal response as depicted in Figure 3. The figure shows that the increase of the *noise* variable in *pop0* leads to an increase in the mean-firing rate, which is the source variable for the BOLD monitor (Figure 3A, blue line). This increase of activity results in an increase of the input signal (input variable *I_CBF*) of the BOLD model depicted in Figure 3B, consequently leading to an increase of the BOLD signal depicted in Figure 3C. After resetting the *noise* variable, the firing rates of both populations reach again the same level, which reduces the input signal of the BOLD model as well as the resulting BOLD signal.

### 3.4. BOLD Model Definition

In the previous example, the default Balloon model (*balloon_RN*) was used as the BOLD model, but ANNarchy allows users to create their own BOLD model by defining a *BoldModel* object representing the desired equations. We describe the definition of a *BoldModel* object using the BOLD model *balloon_RN* (described in Section 2, applied in Section 3.3) as an example. This BOLD model is implemented as follows:


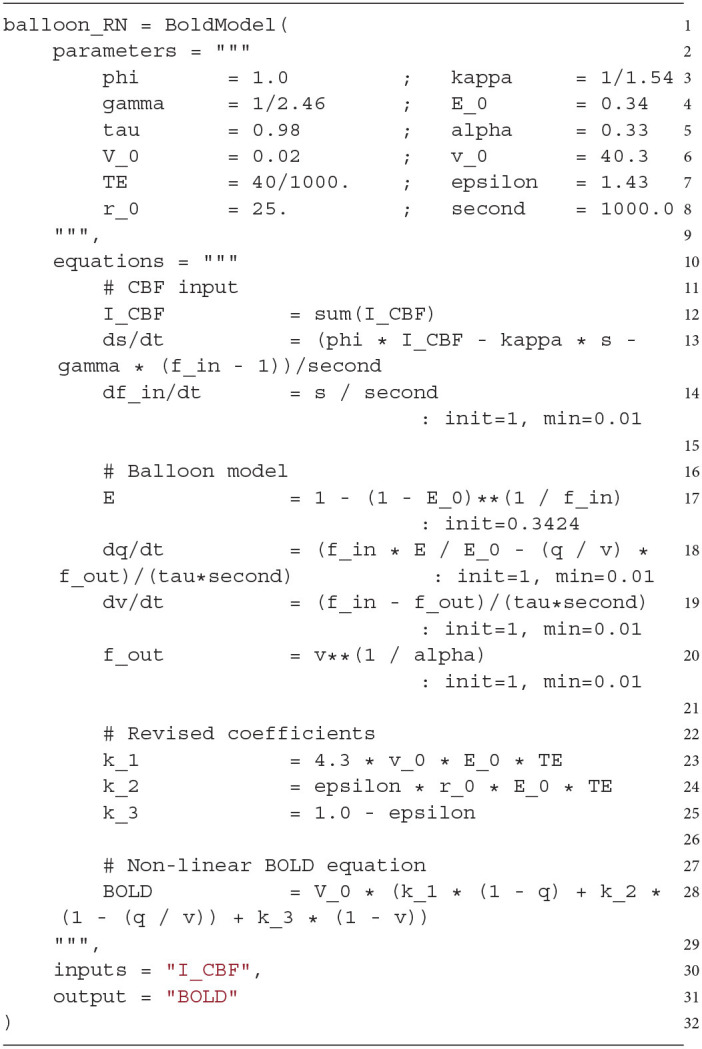


A *BoldModel* object requires a *parameters* argument (line 2), which is a string defining all constants of the BOLD model in a key-value pair notation, i.e., a parameter name on the left and the initialization value on the right side of the assignment operator.

The *equations* argument (line 10) describes all time-dependent variables, defined either by regular equations or ordinary differential equations evaluated on a fixed time grid. Note that parameters resulting from the combination of other parameters can also be defined here (in this example *k_1, k_2, k_3*). In the case of a regular equation, the variable name is on the left side and the update performed in each step on the right side. If the update is defined by a differential equation, the left side needs to contain a $\frac{d\left[var\right]}{dt}$ symbol. To limit the range of values taken by a variable, the *min* and *max* keywords can be used. The initial value for variables is 0.0 by default, but it can be changed by providing an *init* keyword.

The *inputs* argument (line 30) specifies which input signals are expected by the BOLD model. It consists of a single string or a list of strings. These variables can be accessed in the BOLD model definition by using *sum(NAME)* in the *equations* argument, where *NAME* corresponds to the name of the variable (here *I_CBF*, line 12).

Finally, in the *output* argument (line 31), one output variable of the BOLD model is defined, which is automatically recorded by the BOLD monitor. In the following implementation example and all other BOLD models implemented in ANNarchy mentioned in this work, this default output variable corresponds to the BOLD signal (variable *BOLD*), which is also the default value for the *output* argument (here only defined for demonstration purposes).

The *balloon_RN* model is one of the four pre-implemented BOLD models (*balloon_RN, balloon_RL, balloon_CN* and *balloon_CL*, Stephan et al., 2007) and therefore does not need to be defined by the user (but its parameters can be changed dynamically). With the *BoldModel* object, the user can implement new models with the same equation-based interface. For example, a user might want to additionally implement the Davis model (Davis et al., 1998) described by Equation 1 to calculate the change of the BOLD signal Δ*BOLD* from normalized CBF *f* and CMRO2 *r*.


(1) $$\begin{array}{l} \Delta BOLD=M\left[1-f^{\alpha }{\left(\frac{r}{f}\right)}^{\beta }\right] \end{array}$$

Here, *M*, α, and β are additional parameters of the Davis model. In the *BoldModel* above, the normalized CBF is already defined (*f*_*in*_). Thus, only the calculation of the normalized CMRO2 (*r*) must be added. This could be done with the term $f_{in}\cdot \frac{E}{E_{0}}$ (see also Buxton et al., 2004). The following code demonstrates how the previous *BoldModel* could be extended to additionally compute the normalized CMRO2 (*r*, line 34) and the Davis model BOLD signal (line 35) in the *equations* argument:


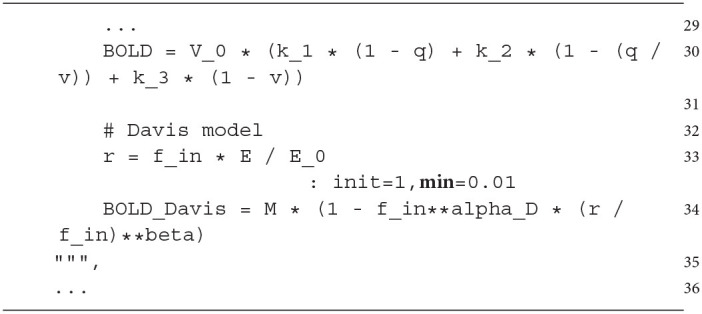


This way, a custom *BoldModel* is obtained, where the BOLD signal is additionally calculated according to the Davis model and the modified signal (*BOLD*_*Davis*_) can additionally be recorded. In addition, the parameters of the Davis model would have to be added to the *parameters* argument, which we have not shown explicitly (but see Supplementary Section 4.3 for a full implementation).

## 4. Example Use Cases

### 4.1. Model Description

**Figure 4:**
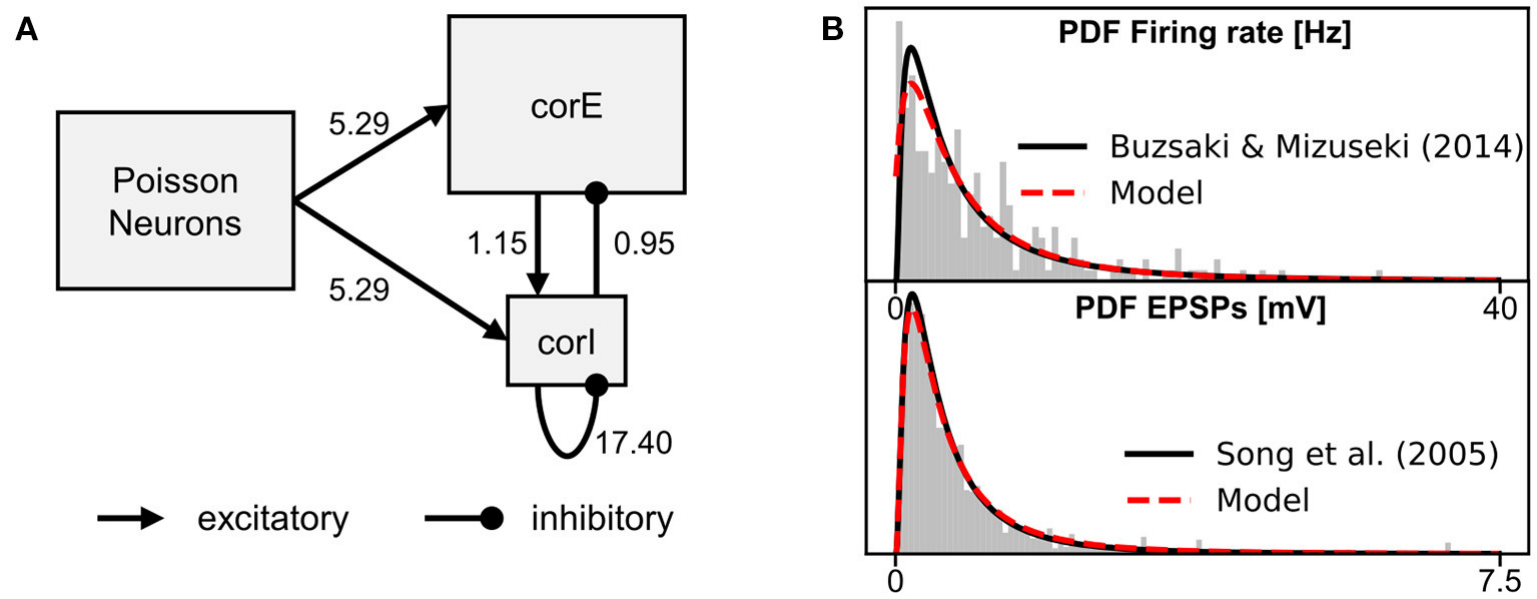
Overview of the microcircuit model. **(A)** The structure of the microcircuit model. Rectangles represent the populations and arrows the projections. The numbers at the projections indicate the factors which scale the weights of each projection. **(B)** Probability density function (PDF) and histogram of the firing rate distribution of the neurons of the corE and corI populations (top) and PDF and histogram of the excitatory post-synaptic potentials (EPSPs) evoked by single spikes in the excitatory and inhibitory neurons (bottom). The weights for the projections of the microcircuit model were drawn from a weight distribution which was tuned to generate the EPSPs distribution of Song et al. (2005) (indicated in black). The weights of each projection were further scaled so that the corE and corI populations produce the firing rate distribution of Buzsáki and Mizuseki (2014) (indicated in black). The scaling factors were optimized, for more details see Supplementary Section 3.3.

In this section, we implement a simple network model of a cortical microcircuit (hereafter referred to as microcircuit model) to further demonstrate use cases of the BOLD monitor. The microcircuit model consists of a population of excitatory neurons and a population of inhibitory interneurons. As neuron models, we use a regular spiking cortical neuron model for the excitatory population (corE) and a fast-spiking cortical interneuron model for the inhibitory population (corI), both introduced in Izhikevich (2007). The two populations receive excitatory inputs from another population whose neurons randomly emit spikes such that their inter-spike intervals correspond to a Poisson process (hereafter referred to as Poisson neurons). The structure of the microcircuit model is shown in Figure 4A. The projections of the microcircuit model include feed-forward excitation (Poisson neurons → corE), feed-forward inhibition (Poisson neurons → corI → corE), and feedback inhibition (corE → corI → corE). The ratio between excitatory neurons and inhibitory interneurons is 4:1, as found, for example, for the visual cortex (Beaulieu et al., 1992; Potjans and Diesmann, 2014). The equations and parameters of the microcircuit model can be found in Supplementary Section 3.

Each neuron receives synaptic input from 10 random neurons in the pre-synaptic population for each projection. Following our previous modeling approaches (Baladron et al., 2019; Goenner et al., 2021; Maith et al., 2021), we model synaptic inputs as conductance-based synapses in our neuron models. Therefore, the synaptic currents (which drive the membrane potential of the neurons) are proportional to the product of a voltage difference (between the membrane potential and the synaptic reversal potential) and a conductance variable representing the spike input of the corresponding synapse (see Supplementary Section 3 for equations). We model only two different types of conductance-based synapses, excitatory synapses (AMPA) and inhibitory synapses (GABA). The conductance variables of the synapses are instantaneously increased by a fixed value (by the weight of the synaptic connection) for each incoming spike and otherwise decay exponentially to zero with a time constant of 10 ms.

A conductance greater than zero causes a synaptic current that drives the membrane potential toward the reversal potential associated with the synapse (0 mV for AMPA synapses and −90 mV for GABA synapses). All synaptic weights are drawn from a log-normal distribution and scaled by a factor for each projection during model initialization. The weights and scaling factors were optimized to replicate distributions from excitatory post-synaptic potentials (Song et al., 2005) and firing rates (Buzsáki and Mizuseki, 2014) with the microcircuit model (see Figure 4B). Further details about obtaining the distributions and optimizing the parameters can be found in Supplementary Section 3.3.

Although the use of neuron models mimicking spiking patterns of real cortical neurons and tuning the parameters to replicate experimental data can provide more realistic network models (see e.g., Humphries et al., 2006; Günay et al., 2008; Pospischil et al., 2008; Goenner et al., 2021), the microcircuit model presented here only aims at demonstrating the application of the BOLD monitor and not at replicating any particular experimental data. To keep the model simple, we chose a network model with two spiking populations and multiple excitatory and inhibitory projections. No particular functional processing takes place in this microcircuit model, as it consists of only two small homogeneous populations, the connectivity is random and synaptic plasticity, important neurotransmitters such as NMDA, the effect of neuromodulators and potential dynamic changes in activity were not taken into account during construction. However, the applicability of the BOLD monitor to larger-scale network models is demonstrated in Section 4.4.

### 4.2. Normalization for Resting-State Activity

We first demonstrate the effect of baseline normalization in the BOLD monitor using the microcircuit model. To do so, we simulate a brief stimulus presentation corresponding to studies of the event-based BOLD response (Glover, 1999; Serences, 2004) by briefly increasing the mean firing rate of the Poisson neurons and meanwhile recording the BOLD response.

All simulations start with an initialization period of 2 s to allow the microcircuit model to enter its steady-state. After that, the recordings are started. A 10-s resting-period is simulated, after which the mean firing rates of the Poisson neurons are increased by a factor of five for 100 ms (hereafter referred to as stimulus pulse). Finally, another post-stimulus resting-period is simulated until a total simulation time of 25 s. This procedure is performed for 40 different random microcircuit model initializations (each with different seeds producing different synaptic contacts, weights, and mean firing rates of Poisson neurons). Additionally, we run 40 simulations without a stimulus pulse, in which only a 25 s resting-period is simulated for comparison.

The BOLD response is recorded simultaneously using two differently initialized BOLD monitors. Both BOLD monitors use the default BOLD model (*balloon_RN*) shown in Section 3.4 and determine the BOLD signal of the ROI which comprises both the corE and corI populations. The source variable for the BOLD monitor is the synaptic activity of the neurons normalized by the number of afferent connections, which has already been used and described in Maith et al. (2021). The key difference between the two BOLD monitors is the baseline normalization. One BOLD monitor uses no baseline normalization and the other BOLD monitor uses a baseline computed over the first 5 s after the 2-s initialization period.

**Figure 5:**
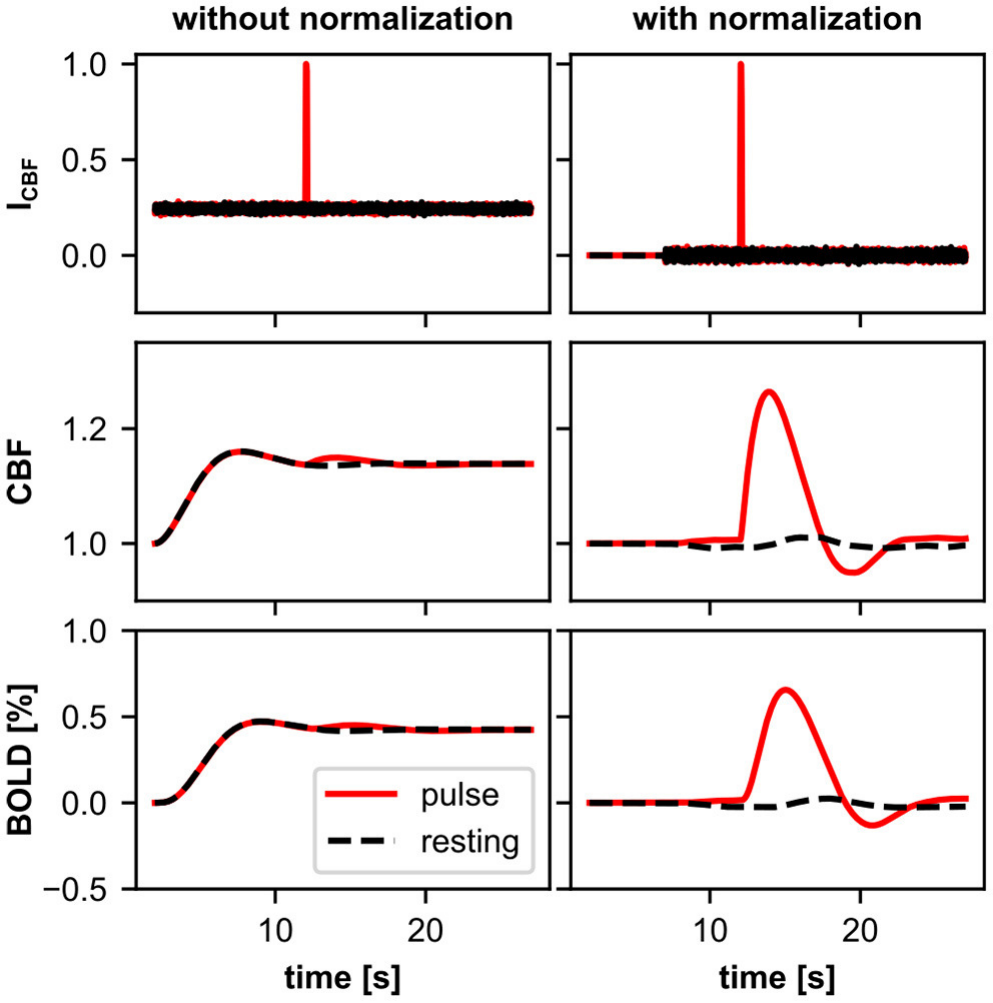
Recordings from two BOLD monitors with (right) and without (left) baseline normalization. Shown are the averaged recordings of 40 resting-state simulations (black) and 40 simulations with a 100 ms stimulus pulse (red). Both BOLD monitors use the same source variable of the same underlying microcircuit model. For the BOLD monitor with baseline normalization, the input signal to the Balloon model (*I*_CBF_) corresponds to the relative change in the signal from the source variable, thus fluctuates around zero. Whereas, without baseline normalization, the input signal has an offset greater than zero. Thus, with baseline normalization only, the normalized CBF signal and the BOLD signal of the Balloon model during the resting-state are approximately one and zero, respectively, corresponding to the definition of the Balloon model. The response to the stimulus pulse is more pronounced with baseline normalization. For visualization, *I*_CBF_ values are shown divided by their maximum value.

Figure 5 shows the recorded variables of the BOLD model: the input variable (I_CBF_) of the BOLD model and the resulting BOLD signal. Although the response of the microcircuit model to the stimulus pulse can be clearly seen in the I_CBF_ of both BOLD monitors, an important difference is that the I_CBF_ of the BOLD monitor without baseline normalization has an offset greater than zero, while the I_CBF_ with baseline normalization fluctuates around zero. It is also noticeable that I_CBF_ with baseline normalization is zero in the first 5 s. This is because the input variable for the BOLD model is not calculated during the time in which the baseline for normalization is determined. In the normalized CBF signal and the BOLD signal, one can clearly see the effect of baseline normalization on the Balloon model dynamics. The response to the stimulus pulse is much more pronounced for the BOLD monitor with baseline normalization. Without baseline normalization, the normalized CBF signal and BOLD signal at rest have an offset greater than zero, whereas with baseline normalization, the signals fluctuate around one and zero, respectively.

The CBF and BOLD signals are defined in the Balloon model relative to their value at rest (normalized CBF and relative change of BOLD). Therefore, the normalized CBF signal or the BOLD signal should only deviate from one or zero, respectively, when the underlying system deviates from its resting-state. For models with resting-state activity, we recommend using the baseline normalization of the BOLD monitor when using the Balloon model.

### 4.3. The Effect of Different Source Variables

One important motivation for developing the BOLD monitor is to provide a simple way to flexibly adjust both the source variables and the BOLD model itself. In Section 3.4, we have already shown how to implement a user-defined BOLD model. Here, we also want to show the possibility to use different variables of the neurons as source variables. The underlying neural processes influencing CBF and CMRO2, and thus ultimately the BOLD signal, are still rather unclear (Howarth et al., 2021). Many different hypotheses and modeling approaches can be found in the literature (Smith et al., 2011; Van Hartevelt et al., 2014; Bennett et al., 2015; Heikkinen et al., 2015; Schmidt et al., 2018). The flexible BOLD monitor in ANNarchy allows us to easily create and compare BOLD models implementing different hypotheses on spiking or rate-coded network models. In this section, we demonstrate this by implementing six different hypotheses using our microcircuit model. For each hypothesis, we add a different BOLD monitor to the microcircuit model, each with different source variables. The six different BOLD monitors are summarized in Table 1. The source code for adding them to the microcircuit model can be found in Supplementary Section 4. Note that the simulated BOLD signals are not compared with experimental data, so we do not make any statements about the validity of the hypotheses. Such an analysis would require an extensive underlying network model, tailored to the brain region under investigation.

**Table 1:** The input and source variables of the 6 different BOLD monitors of Section 4.3.

**Monitor ID**	**BOLD model**	**Input variables**	**Source variables**	
			**corE** **corI**	
A	*balloon_RN*	*I*_CBF_	*syn*	
B	*balloon_RN*	*I*_CBF_	*g*_AMPA_	
C	*balloon_RN*	*I*_CBF_	*r*	
D	*balloon_two_inputs*	*I*_CBF_	*I*_AMPA_+1.5*I*_GABA_	
		*I*_CMRO2_	*I*_AMPA_	
E	*balloon_two_inputs*	*I*_CBF_	*I*_AMPA_+1.5*I*_GABA_	
		*I*_CMRO2_	*I*_AMPA_ *r*	
F	*balloon_two_inputs*	*I*_CBF_	*I*_AMPA_+1.5*I*_GABA_	
		*I*_CMRO2_	$I_{\text{AMPA}}^{\frac{1}{3}}$	

*If the source variables of a specific input variable are different for excitatory and inhibitory neurons (corE and corI populations), they are given separately for corE and corI. I_CBF_, CBF-driving input; I_CMRO2_, CMRO2-driving input; syn, normalized total synaptic activity; g_AMPA_, conductance variable of AMPA synapse; r, neuron firing rate; I_AMPA_, current caused by AMPA synapses; I_GABA_, current caused by GABA synapses*.

**Figure 6:**
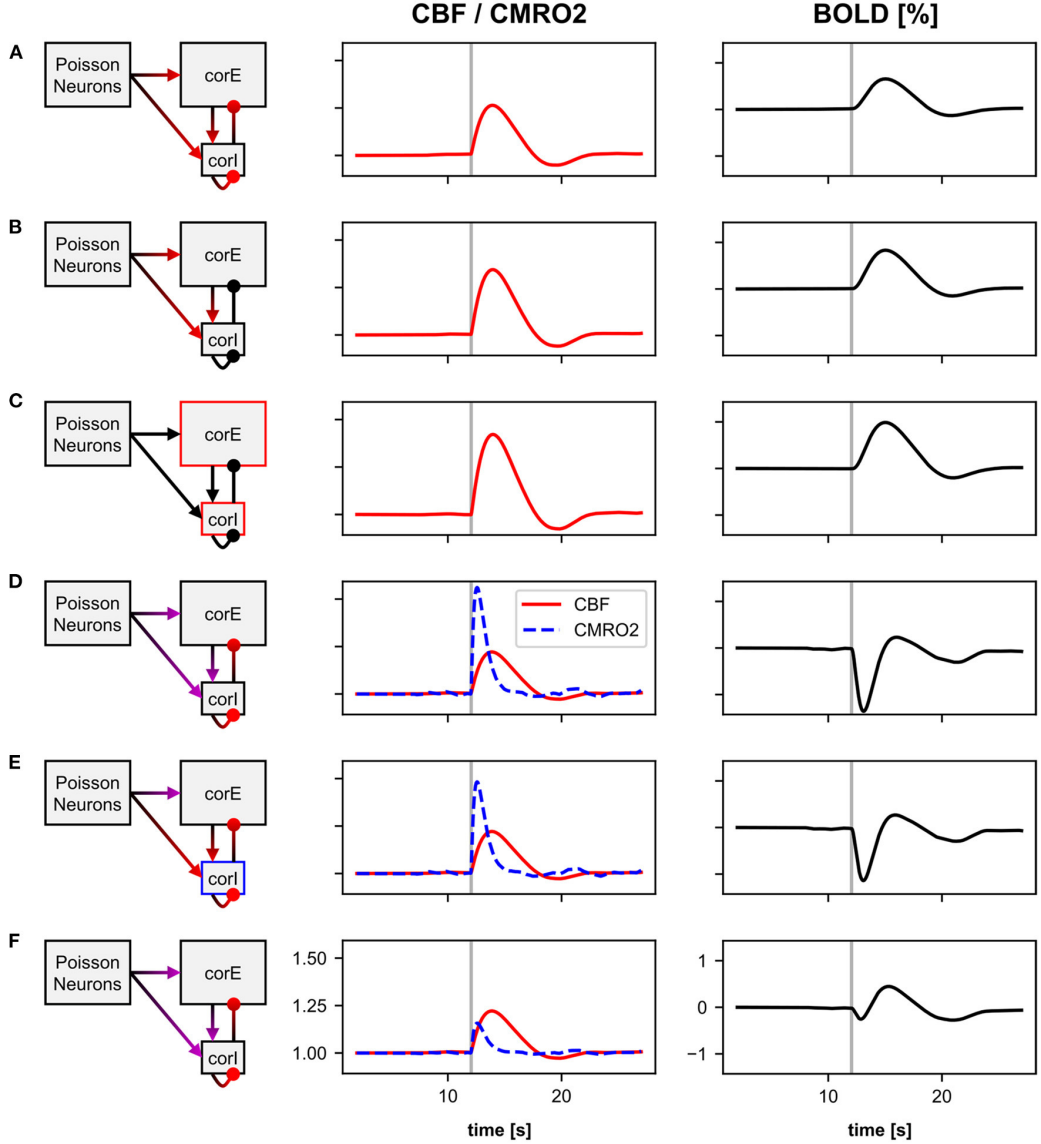
Normalized CBF, CMRO2, and BOLD (relative change) responses of six different BOLD monitors including different source variables. The averaged recordings of 40 simulations with a 100 ms stimulus pulse are shown. The vertical gray bar in columns two and three indicate the time window of the active stimulus pulse. The left column illustrates, which compartments of the microcircuit model were used as source variables for the BOLD monitors. red, CBF-driving; blue, CMRO2-driving; purple, CBF and CMRO2-driving. For a more detailed description about the source variables see Section 4.3 and Table 1. The BOLD monitors **(A–C)** using the classic Balloon model with a single CBF-driving input signal. The BOLD monitors **(D–F)** using the two-input Balloon model defined in this work with two input signals driving CBF and CMRO2 separately.

We again use the stimulus pulse simulation from Section 4.2 to compare the different BOLD signal responses (see Figure 6). The first three hypotheses are based on previous studies that used the classic Balloon model. Thus, we also use the classic Balloon model (BOLD model *balloon_RN*) for the BOLD calculation, which includes a single CBF-driving input signal (see Figure 1A) whose source variable we vary for each hypothesis. The first hypothesis we implement is that the CBF or the BOLD signal is driven by the total synaptic activity of the neurons (as in Van Hartevelt et al., 2014; Schmidt et al., 2018; Maith et al., 2021). To implement this, we use the normalized synaptic activity as the source variable of the BOLD monitor (BOLD monitor A), as previously in Section 4.2. The second hypothesis we implement is that the CBF or the BOLD signal is driven only by the excitatory (glutamatergic) synaptic activity (similar to Heikkinen et al., 2015). For this, we use the conductance variable of the excitatory synapses of the neurons as source variable for the BOLD monitor (BOLD monitor B). The third hypothesis is that the CBF or the BOLD signal is driven by the neuronal output of the neurons, for example, the mean firing rate (as in Smith et al., 2011; Bennett et al., 2015). Thus, for this BOLD monitor (BOLD monitor C), we use the mean firing rate of the neurons as source variable, as in Section 3.3. Figures 6A–C shows that the normalized CBF and BOLD responses vary for these three BOLD monitors with different source variables. The response based on the mean firing rates (Figure 6C) is the strongest, because the firing rates change more relatively to the resting-state compared to the two other source variables. However, the shape of the responses is almost identical.

As mentioned in Section 2.2, it has also been proposed that the CBF and CMRO2 are driven in parallel in a feed-forward manner. Therefore, for the following three BOLD monitors, we use the two-input Balloon model defined in Section 2.2 (*balloon_two_inputs*), which requires two input signals (*I*_CBF_, *I*_CMRO2_, see Figure 1). The source variables used to obtain *I*_CBF_ and *I*_CMRO2_ can be freely chosen from the neuron models of the corE and corI populations.

The first hypothesis considering CBF and CMRO2 being driven in parallel proposes that the CMRO2 is driven only by excitatory synaptic processes and that the CBF is driven by both excitatory and inhibitory synaptic processes (Buxton, 2012, 2021). To implement this hypothesis in BOLD monitor D, we define the current caused by AMPA synapses (*I*_AMPA_) as the CMRO2-driving source variable, and the sum of *I*_AMPA_ and the current caused by GABA synapses (*I*_GABA_) as the CBF-driving source variable. These source variables have to be additionally defined in the neuron models of the neurons of the corE and corI populations (see Supplementary Section 4.1).

The next hypothesis is similar, but additionally states that in inhibitory interneurons, energy consumption, and thus CMRO2, is driven by neuronal output rather than synaptic input (in contrast to excitatory neurons) (Howarth et al., 2021). To implement this in BOLD monitor E, the mean firing rate of the neurons rather than *I*_AMPA_ is defined as the CMRO2-driving source variable for the inhibitory interneurons of the corI population. For the excitatory neurons of the corE population, the same source variables are used as in the previous BOLD monitor.

Figures 6D,E show that the normalized CBF, CMRO2, and BOLD (relative change) responses of these two BOLD monitors are significantly different from the previous ones (with a single input). The BOLD signal shows a much stronger initial dip as CMRO2 increases much faster than CBF. There is little difference between the responses of the two BOLD monitors. The CMRO2 of BOLD monitor E is slightly lower because the firing rate of the inhibitory interneurons increases less than their synaptic current caused by AMPA synapses. However, because the inhibitory interneurons only contribute one-fifth to the input signal of the BOLD monitor (due to the ratio between corE and corI sizes), there is only a small difference from BOLD monitor D to E.

In the last BOLD monitor (Figure 6F), we use almost the same source variables as in BOLD monitor D. We only introduce an additional non-linear operation for the source variable driving CMRO2 by defining the current caused by the AMPA synapses, to the power of one third, as the source variable (instead of the current itself). As a result, energy consumption or CMRO2 no longer increases linearly with the current. Thus, we are still basically following the same general hypothesis (CMRO2 driven by AMPA synaptic processes, CBF driven by AMPA and GABA synaptic processes), but assuming different mathematical relationships for CMRO2. This change causes CMRO2 to increase much less due to the stimulus pulse, as shown in Figure 6F. Thus, the initial dip in the BOLD response is smaller than for the BOLD monitors D & E.

In summary, the BOLD monitor allows users to determine the BOLD signal based on individually chosen source variables. Without much effort, we can define different source variables and even compare different BOLD models (e.g., a model with two input variables). With the classic Balloon model, the BOLD response for our microcircuit model hardly differs for different source variables. Since all variables in the microcircuit model increase similarly in response to the stimulus pulse, the BOLD response also looks similar and only differs in amplitude. When driving CBF and CMRO2 in parallel with different source variables, the choice of the source variable is much more important, because the relationship between them critically affects the shape of the BOLD response not only the amplitude. Nevertheless, the effect of changing the source variable on the resulting BOLD signal may be different for other underlying network models with different dynamics of the different variables (e.g., synaptic currents, mean firing rate, etc.), even when the classic Balloon model is used.

**Figure 7:**
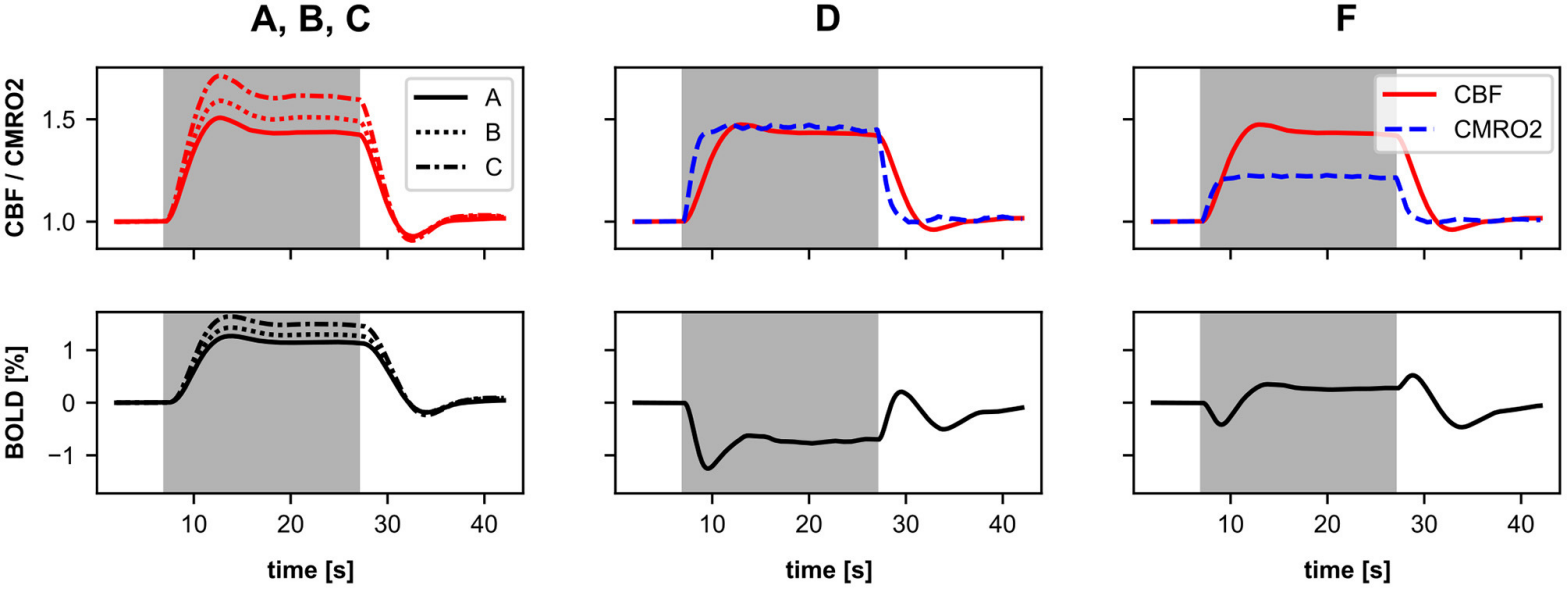
Normalized CBF, CMRO2, and BOLD (relative change) responses of the different BOLD monitors (**A–C**, left), (**D**, mid), and (**F**, right) including different source variables. The averaged recordings of 40 simulations with a 20 s stimulus pulse are shown. The background highlighted in gray indicates the time window of the active stimulus. Here, the firing rate of the Poisson neurons is increased by a factor of 1.2 for 20 s. For a more detailed description about the source variables see Section 4.3 and Table 1. The BOLD monitors **(A–C)** using the classic Balloon model with a single CBF-driving input. The BOLD monitors **(D–F)** using the two-input Balloon model defined in this work with separately driven CBF and CMRO2. The responses of BOLD monitor E are shown in the Supplementary Figure 1 as they are almost identical to those of BOLD monitor **(D)**.

In a second experiment, we perform a simulation with sustained stimulation (longer stimulus pulse) with our six different BOLD monitors. The firing rate of the Poisson neurons is increased by a factor of 1.2 for 20 s. Like in the stimulus pulse simulations, the responses of the first three BOLD monitors (A-C) are very similar and only differ in amplitude (Figure 7, left). The CBF or BOLD responses show a slight initial overshoot, then reach a plateau, and finally, show a slight post-stimulus undershoot. The three BOLD monitor variants with two input signals (D-F) again show significant differences from the three BOLD monitors using the classic Balloon model. The BOLD monitors D and E showed almost identical responses consisting of an initial undershoot a negative plateau and a post-stimulus over- and undershoot (for results of BOLD monitor E see Supplementary Figure 1). It is particularly noticeable that the plateau is negative for the BOLD monitors D & E but not for BOLD monitor F because only for BOLD monitor F, the CBF increases more than the CMRO2. This again illustrates how critical the choice of source variables is when CBF and CMRO2 are driven in parallel by them.

### 4.4. Computational Time Analysis

In this section, we study the additional computational time introduced by the BOLD monitor (hereafter referred to as computational overhead). We use a scaled version of the microcircuit model described in Section 4.1, by incrementally increasing the number of neurons for the populations and leaving the number of synaptic inputs for a neuron fixed to 10 connections (from 10 different neurons of the pre-synaptic population) per projection. Table 2 shows an overview of the total number of neurons and connections for each network model instance.

**Table 2:** Overview on the network model sizes used for the computational time analysis.

**Number of**	**Number of**	**Number of**
**recorded neurons**	**neurons**	**connections**
250	450	5,500
500	900	11,000
1,000	1,800	22,000
2,000	3,600	44,000
4,000	7,200	88,000
8,000	14,400	176,000
16,000	28,800	352,000
32,000	57,600	704,000

*The first column are the number of recorded neurons (i.e., corE and corI populations), the second column the number of all neurons (additionally the Poisson population) within the network model and the third column the overall number of connections*.

**Figure 8:**
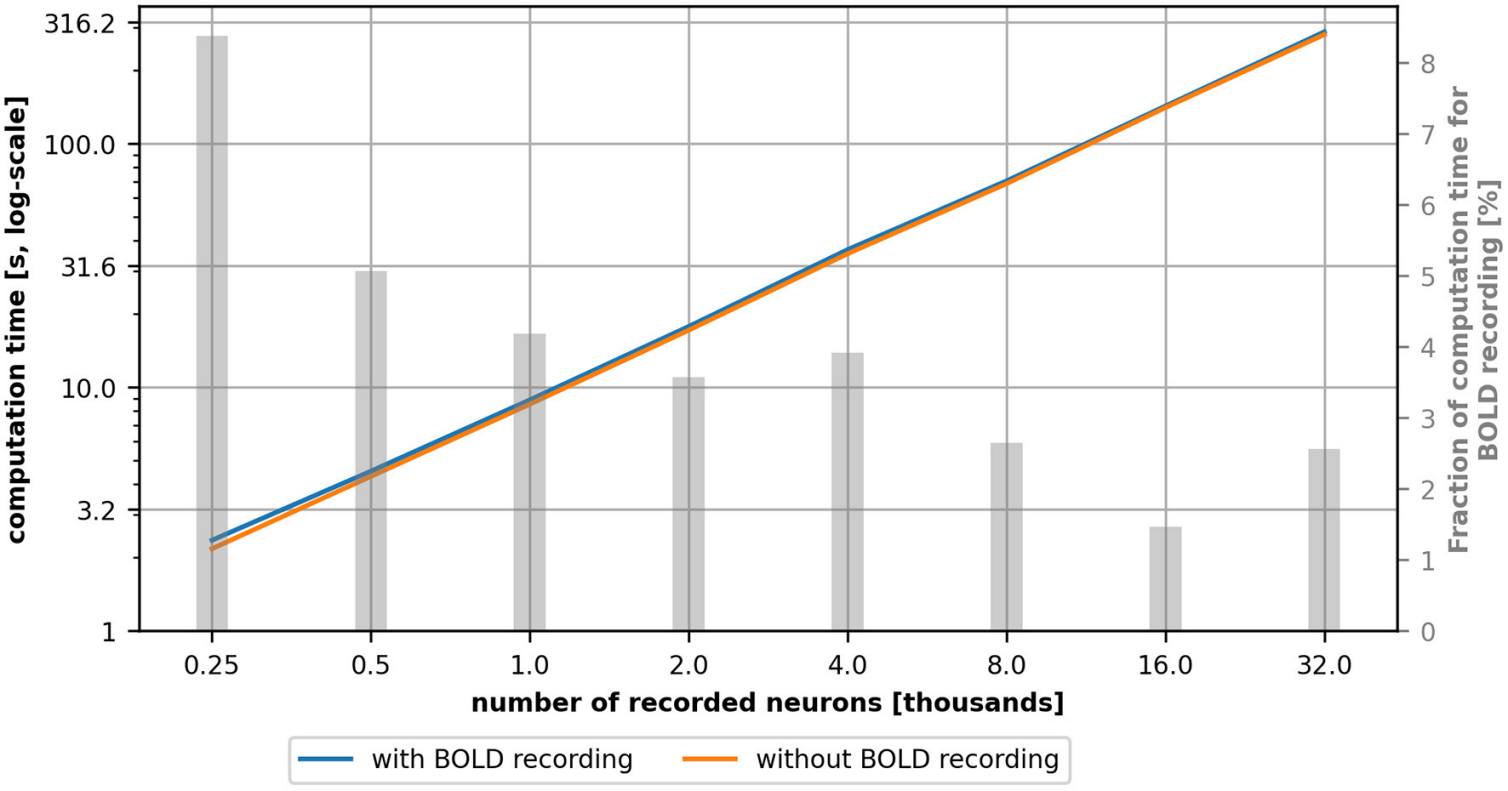
Computational time for a simulation with (blue) and without (orange) BOLD recording as a function of the number of recorded neurons. The gray bars indicate the percentage of the computational overhead of the BOLD recording in the total computation time. This value was computed on the average values and therefore no standard deviation is depicted. The computational overhead is higher for small network models and gets smaller for larger models.

Figure 8 depicts the single thread computational time in seconds as a function of the number of recorded neurons with (blue line) and without (orange line) BOLD recording. For each configuration, we performed 10 runs, each simulating 25 s biological time and we measured the elapsed real time with the Python *time* module. The relative standard deviation was in the range of 0.55% to 2.53% which is too small to be depicted meaningfully in the graph and was therefore omitted.

For all simulated configurations, the computational time with and without BOLD recording is globally similar (between 1% and 8% of overhead depending on the model's size). The relative computational overhead (visualized as gray bars) is larger for small network models but shrinks when the model size increases. Therefore, if network models get more complex, in the sense of number of neurons, complexity of neuron models and the number of connections, one can expect that the share of the computational overhead will shrink accordingly. Overall, the computational time is dominated by the complexity of the network model and the BOLD recording plays a minor role, especially in complex network models.

## 5. Discussion

In this work, we presented a BOLD monitor for obtaining simulated BOLD signals from spiking or rate-coded network models in the ANNarchy neural simulator. All variants of the Balloon model summarized by Stephan et al. (2007), thus the currently prevailing BOLD models, are available as built-in models. The integrated BOLD monitor makes it easy for users to connect their network models to a mathematical BOLD model such as the Balloon model (Buxton et al., 1998) or their own user-defined BOLD models. Users only need to specify from which populations they want to record the BOLD signal, which BOLD model they want to use and which variables of the neurons should be mapped to the input signal(s) of the BOLD model.

The optional baseline normalization of the source variables is a useful feature, as it allows the use of variables with arbitrary magnitudes for the BOLD calculation (including, for example, negative membrane potentials or large synaptic currents), since it sets the relative change of the source variables as the input signal for the BOLD calculation. This is a simple and effective alternative to the previous normalization approaches of the input signals (Schmidt et al., 2018; Maith et al., 2021). Another advantage of baseline normalization is that the resulting input signal for the BOLD model is approximately zero at rest and thus suitable for the Balloon model. A limitation is that it can only use variables that have a relatively constant non-zero mean in the resting-state of the network model. It is highly recommended that users verify that the normalization is appropriate for their chosen variables and used BOLD model. For example, an unsuitable source variable would be the mean firing rate of neurons that are quiescent during the baseline calculation phase and are activated due to a model manipulation after the baseline calculation phase (e.g., during an experiment with input presentation). Another example would be if the selected source variable must first enter a steady-state at the beginning of the simulation (e.g., increase from 0 to a constant non-zero value) and one conducts the baseline calculation during this ramp-up period. This would lead to a too low baseline and thus to a permanently positive normalized signal during recording.

The implementation of the BOLD monitor is flexible enough so that the source variables for the BOLD calculation can be any of the variables present in the neuron models (e.g., a combination of different synaptic currents). Recently, an energy-dependent leaky integrate-and-fire neuron model has been developed that accounts for the neuron's energy consumption by calculating adenosine triphosphate (ATP) dynamics (Jaras et al., 2021). The variables involved there, which are associated with the brain's metabolism, could be of great interest for calculating the BOLD signal and could be easily linked to BOLD models in ANNarchy using the BOLD monitor. Such flexibility makes ANNarchy with the BOLD monitor an useful environment for investigating hypotheses about the coupling between neural processes and BOLD signals, which is an active area of research (Buxton, 2021; Howarth et al., 2021). Since the coupling between neural processes and BOLD signals is still quite unclear, there is no recommended standard method for obtaining simulated BOLD signals with network models (Einevoll et al., 2019). We have demonstrated here how to use the BOLD monitor to study the role of different source variables in a simple network model of a cortical microcircuit. As such, ANNarchy and the new BOLD monitor can support research in neurovascular coupling, which may lead to the development of better BOLD models in the future and possibly to a better understanding of the BOLD response.

The ability to easily obtain BOLD signals from network models opens up more potential applications for ANNarchy, particularly in the area of model-based analysis of neuroimaging data (see Popovych et al., 2019 for a review). The basic idea here is to adjust network models to replicate experimental MRI data while simulating underlying neural processes that cannot be inferred from the MRI data alone. Especially for the study of neuronal diseases in humans, model-based analysis offers new opportunities. Network models customized to patients can be compared with network models customized to healthy controls, or the customized network models can be used as a virtual test bed for specific treatments (Cabral et al., 2013; Van Hartevelt et al., 2014; Jirsa et al., 2017; Meier et al., 2021). Since this approach has been mainly performed with macroscopic network models, ANNarchy can extend this approach by being used mainly in the study of processes at the mesoscopic level of detail. A possible application would be the study of deep brain stimulation (DBS) in, e.g., Parkinson's disease patients, the mechanisms of which may be more extensively and realistically implemented in ANNarchy (similar to other mesoscopic network models, e.g., Rubin and Terman, 2004; Hahn and McIntyre, 2010) than in macroscopic network models (e.g., Meier et al., 2021). Similar to the recently proposed approach to predict DBS-induced clinical improvements using MRI data from Parkinson's disease patients (Horn et al., 2017, 2019), predictors for clinical improvements could also be obtained from model-based analysis of the MRI data. Speculatively, these predictive approaches could potentially even be used in combination with intraoperative MRI (Cui et al., 2016) in the future to optimize electrode positions during DBS electrode implementation. In addition, model-based analysis of MRI data could potentially provide new biomarkers for mental disorders for which MRI data alone are not well-suited (Linden, 2012).

The BOLD monitor is already quite flexible and user friendly, but a potential improvement may be an optional delay for the input signals of the BOLD model. This was demonstrated, for example, for the Balloon model in Buxton et al. (2004). A delayed CBF relative to the CMRO2 could be the cause for the initial dip in the BOLD signal (Buxton et al., 2004; Buxton, 2012). In our two-input Balloon model, we currently implement this with a faster responding for CMRO2 than for CBF. However, whether the initial dip in the BOLD response is actually caused by a faster CMRO2 response is still a matter of debate in the literature (Buxton, 2012). Another useful extension would be individual scaling factors for each source variable signal in the preprocessing of the BOLD monitor. This would allow, for example, one population to be heavily weighted for CBF and another population for CMRO2. Currently, the scaling factor is based on the size of the population and can optionally be adjusted. One of the most important possible further developments concerns the simulation of realistic noise components of the BOLD signal. Experimentally collected BOLD signals are subject to physiological noise, especially motion, cardiac, and respiratory artifacts, as well as instrumental noise (Birn et al., 2008; Chang et al., 2009; Caballero-Gaudes and Reynolds, 2017). To meaningfully compare simulated and experimental signals, these noise sources should also be considered.

We have demonstrated the properties of the BOLD monitor using a simple network model of a cortical microcircuit. However, we did not focus on a use case that includes a comparison of a realistic network model with experimentally obtained BOLD data. Our microcircuit model is not such a use case, but mainly functions as a means to demonstrate the possibilities of the BOLD monitor. Thus, the simulated BOLD responses should not be overinterpreted. Our implementation could be helpful for researchers to compare different BOLD models. Our simulations showed that different source variables of the same underlying network model can affect the simulated BOLD signal differently and, most importantly, that this can be easily tested with the BOLD monitor in ANNarchy. To actually link experimental BOLD signals to their underlying neural processes, more realistic and detailed network models should be used (Vanni et al., 2015).

In this work, we implemented a modified version of the Balloon model in which CBF and CMRO2 are driven in parallel by two different input signals. This two-input Balloon model was composed of model components from previous publications (Buxton et al., 1998, 2004; Friston et al., 2000). By implementing this BOLD model, we demonstrated how ANNarchy allows users to define their own systems of equations as a BOLD model. A BOLD model considering parallel excitation of CBF and CMRO2 will be necessary for future model-based investigation of current hypotheses regarding the origin of the BOLD signal (Buxton, 2021).

Other modeling tools also provide the ability to simulate BOLD signals or analyze MRI data in a model-based manner. One of the best known is Dynamic Causal Modeling (DCM) by Friston et al. (2003), which is included in the Matlab Software Package SPM (Penny et al., 2011). DCM can be used to obtain the effective connectivity of network models from MRI data. The model implementation in DCM differs significantly from that in ANNarchy, where more complex network models can be implemented at finer scales, for example with spiking neurons, detailed neuron and synapse definitions. In DCM, the focus is not on explicitly implementing neural processing, but on investigating how brain regions interact: the dynamics of the brain regions are usually simulated by an activity vector which depends on a connectivity matrix and driving and modulating inputs defined by an experimental paradigm. The length of the activity vector usually corresponds to the number of regions included, i.e., each region is described by one activity value. Simulated BOLD signals for the different brain regions are obtained from the activities of the regions using the Balloon model versions of Stephan et al. (2007). Based on this, free parameters (e.g., the connectivity matrix) are optimized using Bayesian inference to replicate the MRI data and keep the model complexity low (also called Bayesian model inversion). In DCM, other BOLD models than the Balloon model are not available. DCM is not designed to flexibly test hypotheses regarding neurovascular coupling. Therefore, DCM in SPM and ANNarchy with the new BOLD monitor are designed for different applications.

Another modeling tool that incorporates simulation of BOLD signals is The Virtual Brain (TVB) (Ritter et al., 2013; Sanz Leon et al., 2013). TVB is a neural simulator to create large-scale network models usually of the whole cortex and not a mathematical setup for model inversion using BOLD data as DCM, which is only one possible application of TVB. In TVB, network models are usually implemented as a combination of neural mass models, sets of equations that describe the average dynamics of large neuron populations (macroscopic models), but neglect processes at the single-neuron level. Therefore, a TVB – multi-scale co-simulation toolbox that links TVB and neural simulators which model the lower scale processes such as ANNarchy and NEST (Gewaltig and Diesmann, 2007), has been recently introduced (Meier et al., 2021; Schirner et al., 2022). BOLD simulation in TVB is mainly used to validate large-scale network models on experimental MRI data. In TVB, the different versions of the Balloon model of Stephan et al. (2007) are available. However, a flexible definition of source variables or the BOLD model is not currently available because the focus is not on examining the relationship between the BOLD signal and detailed neural processes.

Several successful neural simulators, such as NEST (Gewaltig and Diesmann, 2007) and Brian2 (Stimberg et al., 2019), do not yet have an integrated BOLD simulation routine. For these simulators, users currently have to use external tools for BOLD simulation like the R package neuRosim (Welvaert et al., 2011). Several hemodynamic response functions (HRF) are available in neuRosim, including the Balloon model from Buxton et al. (2004), which can be used to calculate a BOLD response from a given stimulus signal. The stimulus signal typically follows an experimental design, with 1 indicating the presence and 0 the absence of a stimulus. Simulating the BOLD signal based on specific neural processes is actually not the intended use of neuRosim. Nevertheless, neuRosim can be applied to specific simulated signals from network models (Schmidt et al., 2018). A separate definition of the BOLD model (or the HRF in neuRosim) is not currently available. The strengths of neuRosim are the possibility to define spatial positions and the extent of BOLD activation and the modeling of different noise sources of the BOLD signal.

An important advantage of on-line BOLD computation in ANNarchy over off-line computation such as using neuRosim is that simulated data of the recorded neurons (e.g., membrane potentials or synaptic currents) do not need to be stored separately to be used for BOLD computation after simulation. The latter can result in significant increased memory requirements, especially for larger network models. On the other hand, the on-line BOLD computation increases the computation time of the simulations. However, this is a less crucial factor than, for example, the size of the network model, as we show in Section 4.4. Moreover, the share of the on-line BOLD computation in the computation time decreases as the complexity of the model increases. Therefore, the use of the BOLD monitor is also appropriate for larger network models than those used in this work.

In summary, we introduced the BOLD monitor in ANNarchy which allows the on-line computation of simulated BOLD signals directly from spiking or rate-coded network models. Highlights of the BOLD monitor are the flexible definition of source variables in the neuron models of the recorded network model and the possibility to use new user-defined BOLD models. We demonstrated here how this can be done and how this can be used, for example, to compare different hypotheses regarding neurovascular coupling. This tool allows both the validation and optimization of network models with experimental MRI data and the model-based analysis of the BOLD response for a better understanding of its neural basis.

## Data Availability Statement

All used source code is publicly available. This data can be found here: the ANNarchy neural simulator (4.7.0 release) is available on github: https://github.com/ANNarchy/ANNarchy. The simulation code of this work is available on github: https://doi.org/10.5281/zenodo.5547665.

## Author Contributions

OM and HD: designed the research, performed the research, programming, data analysis, and writing (first draft). JB, JV, and FH: guided the research. FH: acquired the funding. OM, HD, JB, JV, and FH: writing (reviewing) and editing. All authors contributed to the article and approved the submitted version.

## Funding

This work was supported by the Deutsche Forschungsgemeinschaft (DFG) SPP-2041 Computational Connectomics as part of the project Clinical Connectomics: A network approach to deep brain stimulation (DFG HA2630/11-2 and HA2630/11-1) and in part by Auto-tuning for neural simulations on different parallel hardware (DFG HA2630/9-1). The publication of this article was funded by Chemnitz University of Technology and by the Deutsche Forschungsgemeinschaft (DFG, German Research Foundation) - 491193532.

## Conflict of Interest

The authors declare that the research was conducted in the absence of any commercial or financial relationships that could be construed as a potential conflict of interest.

## Publisher's Note

All claims expressed in this article are solely those of the authors and do not necessarily represent those of their affiliated organizations, or those of the publisher, the editors and the reviewers. Any product that may be evaluated in this article, or claim that may be made by its manufacturer, is not guaranteed or endorsed by the publisher.
