# Supplementary material for: BOLD Monitoring in the Neural Simulator ANNarchy
Source: Front Neuroinform. 2022 Mar 22;16:790966. doi: 10.3389/fninf.2022.790966 (PMC8981038; doi:10.3389/fninf.2022.790966)
Supplement: Supplementary file 1 [file Data_Sheet_1.pdf]

Supplementary Material

1 IZHIKEVICH NEURON MODEL

The Izhikevich model, used in section 3.3 of the article, is part of the built-in models of the ANNarchy framework. This section shows the equations and parameters used. The equations/parameters are derived from Izhikevich (2003). The model consists of two ODEs, one for the membrane potential v and one for the recovery variable u.

$$\begin{aligned}\frac{dv}{dt} &= 0.04 * v^2 + 5.0 * v + 140.0 - u + I \\ \frac{du}{dt} &= a * (b * v - u)\end{aligned} \tag{S1}$$

As there are no connections in the model the input I consists only of an additive normal noise:

$$I = noise * \mathcal{N}(0.0, 1.0) \tag{S2}$$

The model parameters are chosen according to a regular spiking neuron: $a = 0.02$, $b = 0.2$, $c = -65.0$ and $d = 8.0$.

2 THE BALLOON MODEL

The default BOLD model which is used by the BOLD monitor in ANNarchy is based on the Balloon model version of Stephan et al. (2007) with revised coefficients and a nonlinear BOLD equation (see below for more details regarding these specifications). This Balloon model version is defined by the following equations:

$$\begin{aligned}\dot{s}_{CBF} &= \phi_{CBF}\, I_{CBF} - \kappa_{CBF}\, s_{CBF} - \gamma_{CBF}\,(f_{in} - 1) \\ \dot{f_{in}} &= s_{CBF} \\ E &= 1 - (1 - E_0)^{\frac{1}{f_{in}}} \\ \dot{v} &= \frac{1}{\tau_0}\,(f_{in} - f_{out}) \\ f_{out} &= v^{\frac{1}{\alpha}} \\ \dot{q} &= \frac{1}{\tau_0}\left(f_{in}\frac{E}{E_0} - \frac{q}{v} f_{out}\right) \\ \frac{\Delta BOLD}{BOLD_0} &= V_0\left[k_1\,(1 - q) + k_2\left(1 - \frac{q}{v}\right) + k_3\,(1 - v)\right]\end{aligned} \tag{S3}$$

Here, the normalized CBF (f_{in}) changes as a function of the CBF-driving signal (s_{CBF}), which is driven by an input signal I_{CBF} and subject to negative feedback from itself and f_{in}. f_{in} is coupled to

the blood oxygen extraction fraction (E) and increases the normalized volume fraction of the venous compartment (v), which behaves like a balloon and decreases with outflowing blood (f_{out}). The normalized total deoxyhemoglobin content (q) increases by oxygen extraction of inflowing blood and decreases with outflowing deoxyhemoglobin-containing blood. Finally, relative changes of the BOLD signal ($\frac{\Delta BOLD}{BOLD_0}$) are calculated, based on the nonlinear BOLD equation and the revised coefficients (see below). All parameters which are used by default in the BOLD monitor in ANNarchy for the Balloon model versions of Stephan et al. (2007) are shown in Table S1.

Stephan et al. (2007) summarized four different versions of the Balloon model. They differ in the last equation in the calculation of $\frac{\Delta BOLD}{BOLD_0}$ and the used coefficients in this equation (further called BOLD equation). The coefficients differ between classical and revised coefficients:

$$\begin{aligned} &\text{classical:} \\ &\quad k_1 = (1 - V_0)\, 4.3\, v_0\, E_0\; TE \\ &\quad k_2 = 2\, E_0 \\ &\quad k_3 = 1 - \epsilon \\ &\text{revised:} \\ &\quad k_1 = 4.3\, v_0\, E_0\; TE \\ &\quad k_2 = \epsilon\, r_0\, E_0\; TE \\ &\quad k_3 = 1 - \epsilon \end{aligned} \tag{S4}$$

Regardless of the definition of the coefficients, the parameters from Table S1 are used by default in ANNarchy. Further, Stephan et al. (2007) summarized a linear and nonlinear version for the BOLD equation:

$$\begin{aligned} &\text{linear:} \\ &\frac{\Delta BOLD}{BOLD_0} = V_0\left[(k_1 + k_2)\,(1 - q) + (k_3 - k_2)\,(1 - v)\right] \\ &\text{nonlinear:} \\ &\frac{\Delta BOLD}{BOLD_0} = V_0\left[k_1\,(1 - q) + k_2\left(1 - \frac{q}{v}\right) + k_3\,(1 - v)\right] \end{aligned} \tag{S5}$$

Here, q is the normalized total deoxyhemoglobin content and v the normalized venous volume fraction, both determined in the preceding equations of the Balloon model. The four resulting versions of the Balloon model are given by the following combinations: classical coefficients + linear equation (*balloon_CL*), classical coefficients + nonlinear equation (*balloon_CN*), revised coefficients + linear equation (*balloon_RL*), and revised coefficients + nonlinear equation (*balloon_RN*). These four versions can be used as BOLD

models for the BOLD monitor in ANNarchy. The names of these pre-implemented BOLD models are given in the brackets.

3 CORTICAL MICROCIRCUIT MODEL

3.1 Equations

For the cortical microcircuit model, neuron models from Izhikevich (2007) were used. For the corE population, we use the regular spiking cortical neuron model, shown in Equations S6:

$$\begin{aligned} C\frac{\mathrm{d}v}{\mathrm{d}t} &= k\left(v-v_r\right)\left(v-v_t\right)-u+I \\ \frac{\mathrm{d}u}{\mathrm{d}t} &= a\left(b\left(v-v_r\right)-u\right) \\ \text{if } v \geq v_{peak} \text{ then} &\begin{cases} v & \leftarrow & c \\ u & \leftarrow & u+d \end{cases} \end{aligned} \tag{S6}$$

Here, v is the membrane potential and u the recovery variable. All parameters are shown in Table S2. For the corI population, we use the fast-spiking cortical interneuron model, shown in Equations S7:

$$\begin{aligned} C\frac{\mathrm{d}v}{\mathrm{d}t} &= k\left(v-v_r\right)\left(v-v_t\right)-u+I \\ u_v &= \begin{cases} 0 & \text{if} \quad v < v_b \\ b\left(v-v_b\right)^3 & \text{if} \quad v \geq v_b \end{cases} \\ \frac{\mathrm{d}u}{\mathrm{d}t} &= a\left(u_v-u\right) \\ \text{if } v \geq v_{peak} \text{ then} &\begin{cases} v & \leftarrow & c \\ u & \leftarrow & u+d \end{cases} \end{aligned} \tag{S7}$$

In both neuron models the input current I consists of an excitatory current I_{AMPA} and an inhibitory current I_{GABA} defined by conductance-based synapses. The calculation of I is shown in Equation S8:

$$\begin{aligned} \tau_{AMPA}\frac{\mathrm{d}g_{AMPA}}{\mathrm{d}t} &= -g_{AMPA} \\ \tau_{GABA}\frac{\mathrm{d}g_{GABA}}{\mathrm{d}t} &= -g_{GABA} \\ I_{AMPA} &= -g_{AMPA}\left(v-E_{AMPA}\right) \\ I_{GABA} &= -g_{GABA}\left(v-E_{GABA}\right) \\ I &= I_{AMPA}+I_{GABA} \end{aligned} \tag{S8}$$

Here, the conductances g_{AMPA} and g_{GABA} increase with each incoming spike (from excitatory or inhibitory projections, respectively) by the weight value of the corresponding connection. Otherwise they

decrease exponentially with timeconstants $\tau_{AMPA} = 10$ ms and $\tau_{GABA} = 10$ ms. The reversal potentials are $E_{AMPA} = 0$ mV and $E_{GABA} = -90$ mV.

3.2 Parameters

All parameters of the neuron populations are shown in Table S2.

3.3 EPSPs and firing rate distributions

The weights of the cortical microcircuit model were drawn from a lognormal distribution with mean $\mu = -1.5$ nS and standard deviation $\sigma = 0.93$ nS. The weight distribution was tuned by hand to obtain a distribution of excitatory post-synaptic potentials (EPSPs) similar to the EPSPs distribution from Song et al. (2005) ($\mu = -0.702$ mV, $\sigma = 0.9355$ mV) in corE and corI neurons. To obtain the EPSPs distribution, a single excitatory pre-synaptic neuron was connected to 1000 corE and corI post-synaptic neurons. From a single spike of the pre-synaptic neuron, 2000 EPSPs were obtained, from which the EPSPs distribution was estimated. This was done for different lognormal weight distributions from which the weights of the 2000 connections were drawn until a good visual match was obtained between the EPSPs distribution thus obtained and the distribution from Song et al. (2005). For simplicity, we used the same weight distribution for inhibitory synapses.

To obtain a plausible level of activity in the cortical microcircuit, the weights were additionally scaled. Four scaling factors were introduced for the weights (1) from the Poisson neurons to corE and corI, (2) from corE to corI, (3) from corI to corE, and (4) from corI to corI. The scaling factors were optimized using the Python module hyperopt (Bergstra et al., 2013). The goodness of fit was indicated by the similarity between the firing rate distribution of all cortical neurons (corE and corI) and a target firing rate distribution. The target firing rate distribution (lognormal, $\mu = 1.2$ Hz, $\sigma = 1.1$ Hz) was estimated from distributions presented in Buzsáki and Mizuseki (2014). The distribution of the model was obtained by fitting a lognormal distribution to the firing rates of all cortical neurons using the *scipy* function *scipy.stats.lognorm.fit*. The firing rates were determined over 20-s simulations in which the mean firing rates of the Poisson neurons were drawn directly from the target distribution. To determine the loss for a given set of scaling factors, 20 such 20-s simulations were performed, each with a different randomly initialized model (weights, connections, Poisson rates). For each of these simulations, the mean square error (MSE) between the probability density function of the obtained distribution and the target distribution was determined. The 20 MSEs were averaged to obtain the loss. A total of 10 optimizations were performed with hyperopt and those scaling factors that resulted in the best fit were selected in the end.

4 DIFFERENT BOLD MONITORS

4.1 Initialization of the different BOLD monitors

For the different BOLD monitors used in the main manuscript (Section 4.3), the corresponding source variables must be defined in the neuron models of the corE and corI populations. Thus, the neuron model of the corE population was extended in the equation section by the following lines:

```
tau_syn*dsyn/dt = -syn
var_CBF         = pos(I_AMPA) - 1.5 * neg(I_GABA)
var_CMRO2_a     = pos(I_AMPA)
var_CMRO2_b     = pos(I_AMPA)
var_CMRO2_c     = pos(I_AMPA)**(1/3.)
```

The neuron model of the corI population was extended in the equation section by the following lines:

```
tau_syn*dsyn/dt = -syn
```

```
var_CBF          = pos(I_AMPA) - 1.5 * neg(I_GABA)
var_CMRO2_a      = pos(I_AMPA)
var_CMRO2_b      = r
var_CMRO2_c      = pos(I_AMPA)**(1/3.)
```

Here, *I_AMPA* and *I_GABA* are the synaptic currents of the neuron (see Section 3.1 Equation S8), *syn* is the normalized total synaptic input of the neuron which increases with all incoming spikes (see Maith et al. (2021) for a more detailed description) and decays exponentially with the time constant $\tau_{syn} = 10\,\text{ms}$, and *r* is the averaged firing rate of the neuron, which is determined by ANNarchy over a time window of 20 ms.

These different source variables were used by the six different BOLD monitors. The following code sections show, how the different BOLD monitors were initialized.

BOLD monitor A:

```
1 monA = BoldMonitor(populations=[corE, corI],
2                    normalize_input=5000,
3                    mapping={"I_CBF":"syn"},
4                    recorded_variables=["BOLD", "f_in"])
```

Here, the BOLD signal was computed based on both populations, corE and corI (line 1). The optional baseline normalization was used. The baseline was calculated over the first 5000 ms (line 2). If only one value is given in line 2, the baseline calculation is performed based on the same baseline period for all populations. The synaptic input (variable *syn*) was used as source variable (line 3) which drives the input variable of the BOLD model (*I_CBF*). No BOLD model (argument *bold_model*) was given, thus, the default BOLD model was used which is the Balloon model with revised coefficients and a nonlinear BOLD equation. The variables *BOLD* (the change of the BOLD signal calculated at the end of the Balloon model) and *f_in* (the CBF of the incoming blood) were recorded.

BOLD monitor B:

```
monB = BoldMonitor(populations=[corE, corI],
                   normalize_input=5000,
                   mapping={"I_CBF":"g_AMPA"},
                   recorded_variables=["BOLD", "f_in"])
```

In BOLD monitor B, only the excitatory synaptic activity is used as the source variable. The variable *g_AMPA* represents the conductance of the AMPA synapses of the neurons (see 3.1 Equation S8).

BOLD monitor C:

```
monC = BoldMonitor(populations=[corE, corI],
                   normalize_input=5000,
                   mapping={"I_CBF":"r"},
                   recorded_variables=["BOLD", "f_in"])
```

In BOLD monitor C, again, only the source variable was changed to the averaged firing rate *r*. The variable *r* is available in spiking neurons in ANNarchy without defining it explicitly in the neuron model equations. One has to activate the calculation of the firing rate of the neurons for the corresponding spiking populations corE and corI (function *compute_firing_rate()*).

BOLD monitor D:

```
monD = BoldMonitor(populations=[corE, corI],
                   normalize_input=5000,
                   mapping={"I_CBF":"var_CBF", "I_CMRO2":"var_CMRO2_a"},
                   bold_model=balloon_two_inputs,
                   recorded_variables=["BOLD", "f_in", "r"])
```

In BOLD monitor D we used our self-defined two-input Balloon model (*balloon_two_inputs*, see Section 4.2) as BOLD model. Therefore, one has to link the two input variables of the BOLD model (here *I_CBF* and *I_CMRO2*) which are defined in the corresponding BOLD model equations (Equations S9) with two source variables from the neuron models (here *var_CBF* and *var_CMRO2_a*). The here used coupling causes the variable *var_CBF* to drive the CBF and the variable *var_CMRO2_a* to drive the CMRO2 in the two-input Balloon model. The source variables are described at the beginning of Section 4.1. Here, *I_AMPA* and *I_GABA* drive the CBF and only *I_AMPA* drives the CMRO2 in all neurons, corresponding to recent hypotheses about neurovascular coupling (Buxton, 2021). Additionally to the *BOLD* and *f_in* variable also the *r* variable of the BOLD model was recorded, which is the CMRO2 of the Balloon model.

BOLD monitor E:

```
monE = BoldMonitor(populations=[corE, corI],
                   normalize_input=5000,
                   mapping={"I_CBF":"var_CBF", "I_CMRO2":"var_CMRO2_b"},
                   bold_model=balloon_two_inputs,
                   recorded_variables=["BOLD", "f_in", "r"])
```

In BOLD monitor E the source variable for the CMRO2-driving input variable was changed. Here, CMRO2 is driven by the firing rate in the corI neurons. In the corE neurons CMRO2 is still driven by the variable *I_AMPA* (see beginning of Section 4.1).

BOLD monitor F:

```
monF = BoldMonitor(populations=[corE, corI],
                   normalize_input=5000,
                   mapping={"I_CBF":"var_CBF", "I_CMRO2":"var_CMRO2_c"},
                   bold_model=balloon_two_inputs,
                   recorded_variables=["BOLD", "f_in", "r"])
```

Finally, also in BOLD monitor F the CMRO2 driving variable was changed. Here, the CMRO2 is driven by the variable *I_AMPA* exponentiated by one third, in both populations corE and corI.

4.2 The two-input BOLD model

In the last three BOLD monitors shown in Section 4.1 we used the two-input BOLD model. This self-defined BOLD model is based on compartments of previous Balloon model definitions (Buxton et al., 2004; Friston et al., 2000; Stephan et al., 2007). It allows to drive CBF and CMRO2 in parallel by two separate input signals. It is described by the following equations:

$$
\begin{aligned}
\dot{s}_{CBF} &= \phi_{CBF}\, I_{CBF} - \kappa_{CBF}\, s_{CBF} - \gamma_{CBF}\,(f_{in} - 1) \\
\dot{s}_{CMRO2} &= \frac{\gamma_{CMRO2}}{\gamma_{CBF}}\, \phi_{CMRO2}\, I_{CMRO2} - \kappa_{CMRO2}\, s_{CMRO2} - \gamma_{CMRO2}\,(r - 1) \\
\dot{f_{in}} &= s_{CBF} \\
\dot{r} &= s_{CMRO2} \\
\dot{v} &= \frac{1}{\tau_0}\,(f_{in} - f_{out}) \\
f_{out} &= v^{\frac{1}{\alpha}} + \tau_{out}\,\dot{v} \\
\dot{q} &= \frac{1}{\tau_0}\left(r - \frac{q}{v} f_{out}\right) \\
\frac{\Delta BOLD}{BOLD_0} &= V_0\left[k_1\,(1-q) + k_2\left(1 - \frac{q}{v}\right) + k_3\,(1-v)\right]
\end{aligned}
\tag{S9}
$$

$$
\begin{aligned}
k_1 &= 4.3\, v_0\, E_0\, TE \\
k_2 &= \epsilon\, r_0\, E_0\, TE \\
k_3 &= 1 - \epsilon
\end{aligned}
$$

Here f_{in} is described as a damped oscillator, as introduced by Friston et al. (2000), as in the default Balloon model (Equations S3). Thus, a non-zero input signal (I_{CBF}) causes a change in f_{in}. In a very similar way, a non-zero CMRO2-driving input signal I_{CMRO2} causes a change in the CMRO2 (r). The parameters of the two damped oscillators are chosen so that f_{in} behaves like an underdamped oscillator (to allow overshoot and undershoot) and r behaves like a critically damped oscillator. In addition, the parameters are chosen so that r changes faster corresponding to its input signal I_{CMRO2} than f_{in} to I_{CBF}. By scaling I_{CMRO2} by $\frac{\gamma_{CMRO2}}{\gamma_{CBF}}$, both r and f_{in} reach the same steady-state amplitude with equal input signals. The rest of the model includes the Balloon model equations for v, f_{out}, and q from Buxton et al. (2004). These include an extension for f_{out} compared to the default Balloon model (Equations S3). This extension provides another way to adjust the dynamics of the model through the parameter τ_{out}. In addition, in the equation of q the term $f_{in}\frac{E}{E_0}$ is replaced by r (see also Buxton et al. (2004)). Finally the relative change of the BOLD signal is computed by the nonlinear BOLD equation containing the revised coefficients, as defined by Stephan et al. (2007). The two-input BOLD model is also available as a pre-defined BOLD model in ANNarchy (*balloon_two_inputs*). The default parameters of the two-input BOLD model are shown in Table S3.

4.3 Modified Balloon model with Davis model

In this section, we show the full implementation of the modified BOLD model *balloon_RN* from Section 3.4 of the main script, which additionally includes the Davis model (here called *balloon_RN_Davis*). The parameters used for the Davis model (*M*, *alpha_D*, *beta*) were taken from Griffeth and Buxton (2011).

```
balloon_RN_Davis = BoldModel(
    parameters = """
        phi        = 1.0         ;   kappa      = 1/1.54
        gamma      = 1/2.46      ;   E_0        = 0.34
```

```
        tau         = 0.98        ;   alpha      = 0.33
        V_0         = 0.02        ;   v_0        = 40.3
        TE          = 40/1000.    ;   epsilon    = 1.43
        r_0         = 25.         ;   second     = 1000.0
        M           = 14.9        ;   alpha_D    = 0.14
        beta        = 0.91
    """,
    equations = """
        # CBF input
        I_CBF          = sum(I_CBF)
        ds/dt          = (phi * I_CBF - kappa * s - gamma * (f_in - 1))/second
        df_in/dt       = s / second                                   : init=1, min=0.01

        # Balloon model
        E              = 1 - (1 - E_0)**(1 / f_in)                    : init=0.3424
        dq/dt          = (f_in * E / E_0 - (q / v) * f_out)/(tau*second)  : init=1, min=0.01
        dv/dt          = (f_in - f_out)/(tau*second)                  : init=1, min=0.01
        f_out          = v**(1 / alpha)                               : init=1, min=0.01

        # Revised coefficients
        k_1            = 4.3 * v_0 * E_0 * TE
        k_2            = epsilon * r_0 * E_0 * TE
        k_3            = 1.0 - epsilon

        # Non-linear BOLD equation
        BOLD           = V_0 * (k_1 * (1 - q) + k_2 * (1 - (q / v)) + k_3 * (1 - v))

        # Davis model
        r = f_in * E / E_0                                            : init=1, min=0.01
        BOLD_Davis = M * (1 - f_in**alpha_D * (r / f_in)**beta)
    """,
    inputs = "I_CBF",
    output = "BOLD"
)
```

REFERENCES

Bergstra, J., Yamins, D., and Cox, D. (2013). Making a science of model search: Hyperparameter optimization in hundreds of dimensions for vision architectures. In *International conference on machine learning*, eds. S. Dasgupta and D. McAllester (PMLR), 115–123

Buxton, R. B. (2021). The thermodynamics of thinking: connections between neural activity, energy metabolism and blood flow. *Philosophical Transactions of the Royal Society B* 376, 20190624

Buxton, R. B., Uludağ, K., Dubowitz, D. J., and Liu, T. T. (2004). Modeling the hemodynamic response to brain activation. *Neuroimage* 23, S220–S233

Buzsáki, G. and Mizuseki, K. (2014). The log-dynamic brain: how skewed distributions affect network operations. *Nature Reviews Neuroscience* 15, 264–278

Friston, K. J., Mechelli, A., Turner, R., and Price, C. J. (2000). Nonlinear responses in fmri: the balloon model, volterra kernels, and other hemodynamics. *NeuroImage* 12, 466–477

Griffeth, V. E. and Buxton, R. B. (2011). A theoretical framework for estimating cerebral oxygen metabolism changes using the calibrated-bold method: modeling the effects of blood volume distribution,

hematocrit, oxygen extraction fraction, and tissue signal properties on the bold signal. *Neuroimage* 58, 198–212

Izhikevich, E. M. (2003). Simple model of spiking neurons. *IEEE Transactions on Neural Networks* 14, 1569–1572. doi:10.1109/TNN.2003.820440

Izhikevich, E. M. (2007). *Dynamical systems in neuroscience* (Cambridge: MIT press)

Maith, O., Villagrasa Escudero, F., Dinkelbach, H. Ü., Baladron, J., Horn, A., Irmen, F., et al. (2021). A computational model-based analysis of basal ganglia pathway changes in parkinson's disease inferred from resting-state fmri. *European Journal of Neuroscience* 53, 2278–2295

Obata, T., Liu, T. T., Miller, K. L., Luh, W.-M., Wong, E. C., Frank, L. R., et al. (2004). Discrepancies between bold and flow dynamics in primary and supplementary motor areas: application of the balloon model to the interpretation of bold transients. *NeuroImage* 21, 144–153

Song, S., Sjöström, P. J., Reigl, M., Nelson, S., and Chklovskii, D. B. (2005). Highly nonrandom features of synaptic connectivity in local cortical circuits. *PLoS biology* 3, e68

Stephan, K. E., Weiskopf, N., Drysdale, P. M., Robinson, P. A., and Friston, K. J. (2007). Comparing hemodynamic models with dcm. *Neuroimage* 38, 387–401

Table S1. Default parameters of pre-implemented Balloon model versions from Stephan et al. (2007). The units of the variables are given in the brackets. The third column indicates where the parameter values were taken from. a – Friston et al. (2000), b – Obata et al. (2004)

Parameter	Value	Reference
ϕ_{CBF}	1.0	-
κ_{CBF} $[s^{-1}]$	1.54^{-1}	a
γ_{CBF} $[s^{-2}]$	2.46^{-1}	a
E_0	0.34	a
τ_0 [s]	0.98	a
α	0.33	a
V_0	0.02	a
v_0 $[s^{-1}]$	40.3	b
TE [ms]	40.0	b
ϵ	1.43	b
r_0 $[s^{-1}]$	25.0	b

Table S2. Parameters of the populations corE and corI of the cortical microcircuit model. The units of the variables are given in the brackets. All variables except size are taken from Izhikevich (2007).

Parameter	corE	corI
size	200	50
C [pF]	100.0	20.0
k	0.7	1.0
v_r [mV]	-60.0	-55.0
v_t [mV]	-40.0	-40.0
v_b [mV]	-	-55.0
a	0.03	0.2
b	-2.0	0.025
c [mV]	-50.0	-45.0
d [pA]	100.0	0.0
v_{peak} [mV]	35.0	25.0

Table S3. Default parameters of pre-implemented two-input BOLD model. The units of the variables are given in the brackets. The third column indicates where the parameter values were taken from: a – Friston et al. (2000), b – Obata et al. (2004), c – Stephan et al. (2007), d – Buxton et al. (2004). For τ_{out} there are separate values for a positive/negative $\dot{v}$.

Parameter	Value	Reference
ϕ_{CBF}	1.0	-
κ_{CBF} $[s^{-1}]$	0.77	$0.6\sqrt{4\gamma_{CBF}}$
γ_{CBF} $[s^{-2}]$	2.46^{-1}	a
ϕ_{CMRO2}	1.0	-
κ_{CMRO2} $[s^{-1}]$	4.03	$\sqrt{4\gamma_{CMRO2}}$
γ_{CMRO2} $[s^{-2}]$	0.246^{-1}	$10\,\gamma_{CBF}$
E_0	0.34	a
τ_0 [s]	0.98	a
α	0.33	a
V_0	0.02	a
v_0 $[s^{-1}]$	40.3	b
TE [ms]	40.0	b
ϵ	1	c
r_0 $[s^{-1}]$	25.0	b
$\tau_{out,+}$ [s]	0	d
$\tau_{out,-}$ [s]	20	d

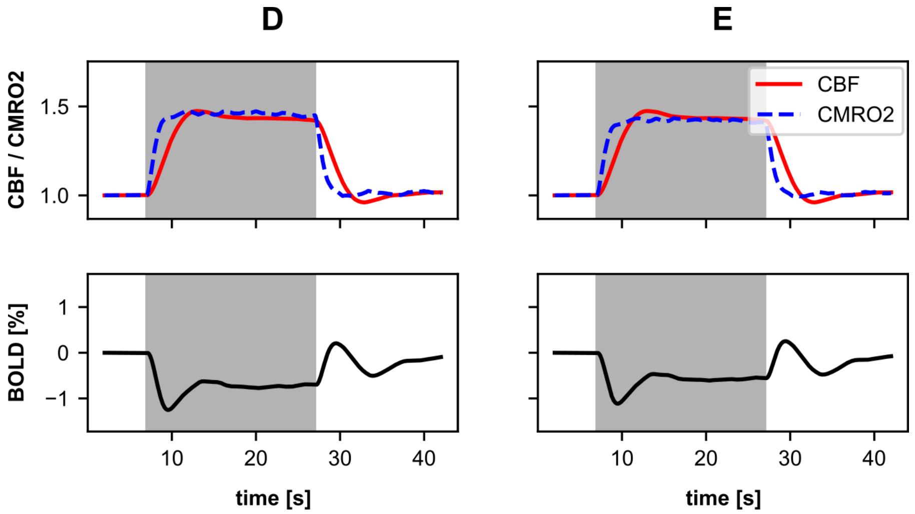

Figure S1. CBF, CMRO2 and BOLD responses of BOLD monitors D and E. Shown are the averaged recordings of 40 simulations with a 20 s stimulus. The background highlighted in grey indicates the time window of the active stimulus. Here, the firing rate of the Poisson neurons is increased by a factor of 1.2 for 20 s. The responses of both monitors are almost identical. In monitor E the CMRO2 increases slightly less because the firing rates of the interneurons (the CMRO2-driving source variable for monitor E) increases less than the current caused by the AMPA synapses (the CMRO2-driving source variable for monitor D). For a more detailed description about the source variables used by the monitors see the main manuscript and Section 4.1.
